# The growth hormone/IGF-1 axis is a risk factor for long-term kidney allograft failure

**DOI:** 10.1172/jci.insight.188485

**Published:** 2025-05-06

**Authors:** Matthew Cusick, Viji Nair, Damian Fermin, John Hartman, Jeffrey A. Beamish, Zeguo Sun, Zhongyang Zhang, Edgar Otto, Rajasree Menon, Sudha Nadimidla, Nicholas Demchuk, Kelly Shaffer, Peter Heeger, Weija Zhang, Madhav C. Menon, Matthias Kretzler, Roger C. Wiggins, Abhijit S. Naik

**Affiliations:** 1 Department of Pathology, and; 2 Department of Internal Medicine, University of Michigan, Ann Arbor, Michigan, USA.; 3 Department of Internal Medicine, and; 4 Department of Genetics and Genomic Sciences, Mount Sinai Hospital, New York, New York, USA.; 5 Department of Transplant Surgery, University of Michigan, Ann Arbor, Michigan, USA.; 6 Cedars Sinai Hospital, Los Angeles, California, USA.; 7 Department of Internal Medicine, Yale University, New Haven, Connecticut, USA.

**Keywords:** Nephrology, Therapeutics, Growth factors, Organ transplantation

## Abstract

**INTRODUCTION:**

Maladaptive hypertrophy, podocyte stress, and depletion contribute to kidney function decline. Although insulin-like growth factor 1 (IGF-1) plays a key role in early hypertrophic responses in the single kidney state, its impact on kidney transplant (KTx) outcomes remains uncertain. This report tests the hypothesis that early IGF-1 exposure reduces KTx survival.

**METHODS:**

Population datasets compared incident death-censored graft failure (DCGF) rates by age at KTx (*n* = 366,404) with IGF-1 levels by age (*n* = 15,014). A clinical study of 216 KTx recipients evaluated the association of IGF-1 exposure with DCGF and secondary outcomes of proteinuria and biopsy-proven acute rejection. IGF-1 exposure was modeled using pre-KTx IGF-1 levels and donor kidney dose estimated from the donor/recipient body surface area ratio reflecting allograft hyperfiltration. The association of DCGF with an *IGF1* SNP linked to high IGF-1 levels was assessed in 724 genotyped allograft recipients. Single-cell transcriptomic data from first-year post-KTx patients and binephric donors were compared to assess intrarenal cellular expression of *IGF1*, *IGF1R*, and growth hormone receptor (*GHR*) transcripts.

**RESULTS:**

DCGF risk by age at KTx paralleled IGF-1 levels by age. Higher IGF-1 exposure was associated with significantly increased risks of DCGF, proteinuria, and T cell–mediated rejection. Genotypic analysis showed a 50% increase in DCGF risk per risk allele at *IGF1* expression quantitative trait locus *rs35767*. First-year biopsy results revealed no increase in intrarenal *IGF1* transcripts, while *GHR* and *IGF1R* transcripts were suppressed, consistent with circulating IGF-1 (vs. graft-derived IGF-1) being the primary source of IGF-1 exposure.

**CONCLUSION:**

We identify a role for the growth hormone/IGF-1 axis in reducing KTx survival.

## Abstract 2

This study investigates the role of Insulin-like Growth Factor-1 (IGF-1) in kidney transplant outcomes, particularly in relation to the donor kidney dose relative to the recipient body size, which also determines hyperfiltration. Higher pre-transplant IGF-1 levels, combined with a lower donor kidney dose, were associated with increased risks of kidney failure, excess proteins in the urine, and transplant rejection. These findings offer critical insights into how modulating IGF-1 levels relative to kidney dose could improve long-term transplant outcomes.

## Introduction

Reduced kidney mass scenarios such as 5/6 nephrectomy, which progresses rapidly, and unilateral nephrectomy (UniNx), which progresses more slowly to kidney failure, develop from adaptations to reduced renal mass (1, 2). These include hyperfiltration driven by nitric oxide–mediated vasodilatation (3) and podocyte hypertrophic stress and depletion, culminating in proteinuria, glomerulosclerosis, and end-stage renal disease (ESRD) (4, 5). Thus, adaptive or maladaptive hypertrophic responses to reduced renal mass can influence long-term kidney outcomes (6, 7).

All single kidney transplants (KTx) hyperfilter and hypertrophy to meet the metabolic demands of the host due to the subnormal kidney mass (dose) typically delivered at transplantation (8). In addition to a single kidney replacing the normal 2-kidney complement, a lower body surface area (BSA) of the donor versus that of the recipient (reflecting a lower kidney dose) is associated with inferior KTx survival (9, 10). Our previous reports also highlight that lower kidney doses are associated with elevated urine markers of podocyte stress, detachment, and accelerated graft function decline, aligning with UniNx studies (4, 5, 11, 12). Notably, podocyte density is significantly decreased by 20% by 3 months and 30% by 12 months after KTx (13). This level of podocyte density reduction in model systems (14) and human studies (15) is associated with glomerular destabilization, glomerulosclerosis, and reduced long-term kidney survival (11–13, 16). Consistent with this hypothesis, even stable allografts have a podocyte detachment rate 6-fold higher than binephric healthy controls (13). Thus, an early hypertrophic but potentially maladaptive response to reduced kidney dose is associated with excess podocyte stress, detachment, and depletion, projected to shorten KTx survival (9, 10, 17–19). Elucidating key mediators driving this maladaptive response could offer targetable candidate pathways to improve long-term KTx outcomes.

The growth hormone (GH) and insulin-like growth factor 1 (IGF-1) axis plays a fundamental role in postnatal body and organ growth (20–22). Data from several studies, including IGF-1 receptor (IGF-1R) blockers, support the concept that IGF-1 also drives the very early kidney growth response to UniNx, which can become maladaptive (23–26). Notably, the rate of remaining kidney growth is maximal by 2 days after UniNx, coinciding with the peak in urinary IGF-1 excretion, simultaneous reduction in circulating IGF-1 level, and increased IGF-1 protein content in remnant kidneys (27–29). However, whether this early kidney IGF-1 exposure results from intrinsic kidney IGF-1 production or comes from the circulation is unclear.

In this report of human KTx, we use population, clinical, genetic data, and single-cell RNA-seq analysis to demonstrate that high-level IGF-1 exposure derived from the circulation is associated with inferior allograft outcomes, comparable to what is observed in partial nephrectomy model systems of kidney failure (1, 4, 5). This hypothesis, that graft exposure to circulating IGF-1 is responsible for driving “compensatory” maladaptations, offers a potentially targetable pathway to extend allograft longevity.

## Results

**Figure 1:**
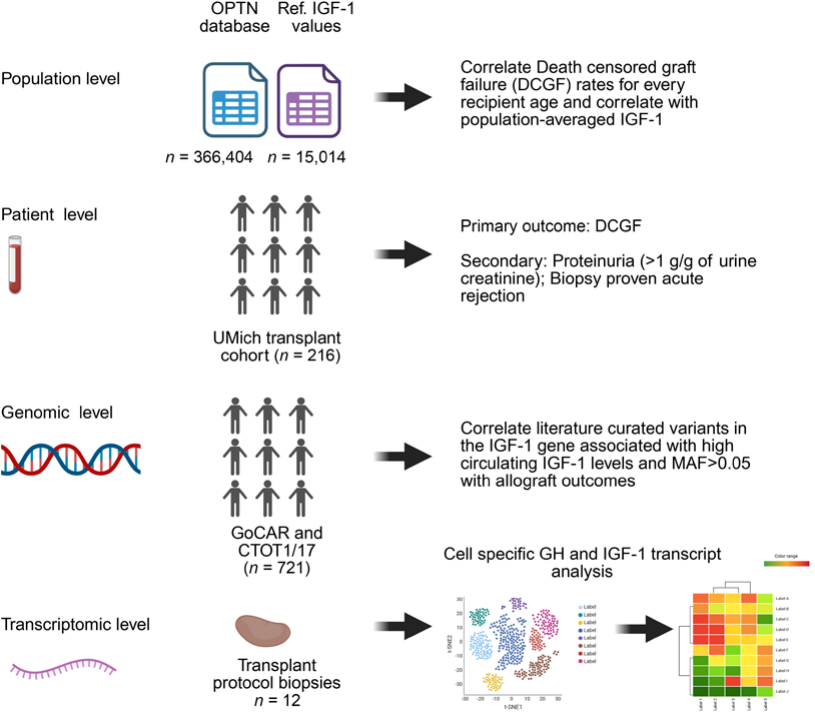
Approaches used to assess the relationship between IGF-1 levels at KTx and long-term allograft survival. Cohort 1: Population-level study. Population-level analysis compared death-censored graft failure (DCGF) rates and time to 50% graft loss among kidney transplant (KTx) recipients 0–60 years of age with IGF-1 levels by age derived from a reference study. Cohort 2: Clinical study. This analysis involved 216 consecutive KTx recipients 0–80 years of age. Pretransplant IGF-1 levels were measured and related to long-term outcomes using survival models. These models were adjusted for donor, recipient, and transplant characteristics, including an estimated kidney dose (eKD) derived from the donor-recipient body surface area ratio (see Results). Adjustments in the Cox survival model included primary outcomes (DCGF), secondary outcomes (proteinuria > 1 g/g), and a composite of alloimmune responses (acute rejection or de novo donor-specific antibody development). Cohort 3: Genotype study. For genetic level analysis, 2 NIH-sponsored studies linking genotype to outcomes in KTx recipients were employed to test the hypothesis that an *IGF1* gene variant associated with high circulating IGF-1 levels correlates with long-term allograft survival. Cohort 4: Transcriptomic study. Our previously published cohort was utilized for transcriptome-level analysis to map IGF system transcript expression in single kidney cells derived from normal kidney allografts with no histologic abnormality in the first year after transplantation, compared to normal kidneys biopsied at the time of transplantation (46).

Figure 1 illustrates the multicohort approaches used to test the hypothesis 

### Cohort 1: population-level study

**Figure 2:**
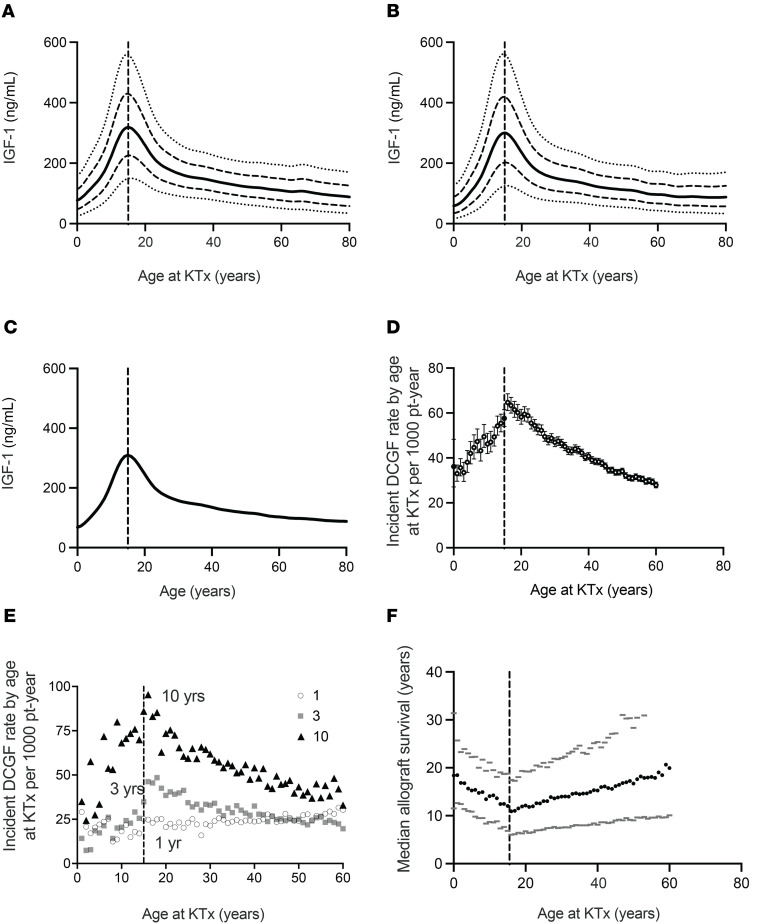
Comparison between 2 reference populations where IGF-1 levels and transplant outcomes by age are tested. (**A**) Age-specific average (solid dark line) IGF-1 levels from Bidlingmaier et al. (30) for males, with 1 SD (dashed lines) above and below mean values. Dotted lines represent the 2-SD values. (**B**) Age-specific average (solid gray line) IGF-1 levels for females, with 1 SD (dashed lines) above and below mean values. Dotted lines represent the 2-SD values. (**C**) Solid line shows mean IGF-1 levels for pooled males and females, which were not statistically significantly different, both peaking at 15 years of age. (**D**) Incident DCGF rate per 1000 patient-years with 95% CI. (**E**) Breakdown of incident DCGF rate per 1000 patient years into short-term (1 year), intermediate-term (3 years), and long-term (10 years) outcomes. (**F**) Median death-censored allograft survival rates in dark circles and their 25% and 75% values in gray. The dashed vertical line represents 15 years of age across all panels.

Table 1 provides the donor, recipient, and transplant characteristics of the Organ Procurement and Transplantation Network (OPTN) cohort. Population-based IGF-1 levels by age were obtained from a report by Bidlingmaier et al. (30). Figure 2, A and B show the mean IGF-1 ± 2SD values for men and women by age in the reference cohort. Figure 2C shows that the mean pooled IGF-1 levels for men and women increase approximately 6-fold from birth, peaking at 15 years and subsequently diminishing into older age. Figure 2D shows the incident death-censored graft failure (DCGF) rate by age at KTx, illustrating that the incident DCGF rate parallels age-related changes in IGF-1 level. Figure 2E shows the incident DCGF rate at 1 year (short-term), 3 years (intermediate-term), and 10 years (long-term), illustrating that at 1 year there was no detectable increase in DCGF in the 15-year-old group. In contrast, at the intermediate (3 years) and especially long-term (10 years) there was an obvious recipient age effect that peaks around 15 years and parallels the peak in age-related IGF-1 levels. Figure 2F shows the time to median allograft survival to illustrate the magnitude of the changes observed, wherein a 15-year-old recipient had a median graft survival of only 12 years. In contrast, in very young allograft recipients and 60-year-old recipients, median graft survival approached 20 years. Thus, comparison of the OPTN and IGF-1 population data shows a strong temporal relationship between IGF-1 level at time of transplant and allograft longevity, and that the major effect of higher IGF-1 level is on intermediate and long-term DCGF risk, but not short-term risk.

#### The era effect.

**Figure 3:**
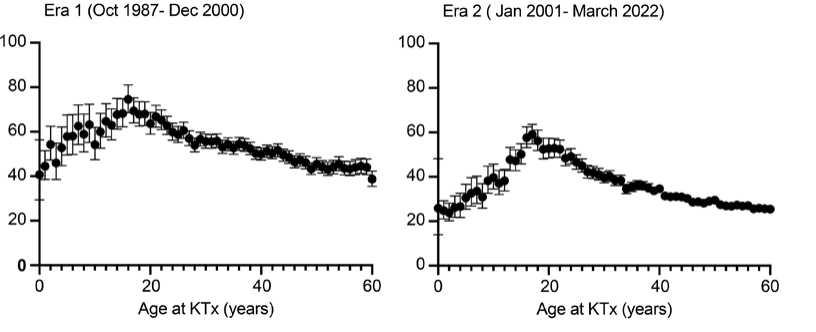
Age-associated trends in death-censored allograft failure rates across transplantation eras and their relationship to IGF-1 levels. (**A**) Incident DCGF rates (per 1,000 patient-years) by age across Era 1 (October 1987–December 2000) and (**B**) Era 2 (January 2001–March 2022) exhibit a similar distribution of failure rates by age at KTx, albeit with lower incidence rates in Era 2. These findings suggest that the relationship between recipient age and incident graft failure rates remains consistent despite advancements in maintenance immunosuppressive regimens and improvements in medical and surgical protocols. Additionally, these trends closely parallel the relationship between IGF-1 levels and age, as illustrated in Figure 2, A–C.

Two time points were selected to represent periods of predominantly cyclosporine (before December 31, 2000) (Figure 3A) and tacrolimus-based therapies (on or after January 1, 2001) (Figure 3B). While the DCGF rate (expressed as incident rate per 1000 patient-years) decreased, the relationship between recipient age and DCGF rates remained similar.

### Cohort 2: clinical study

**Figure 4:**
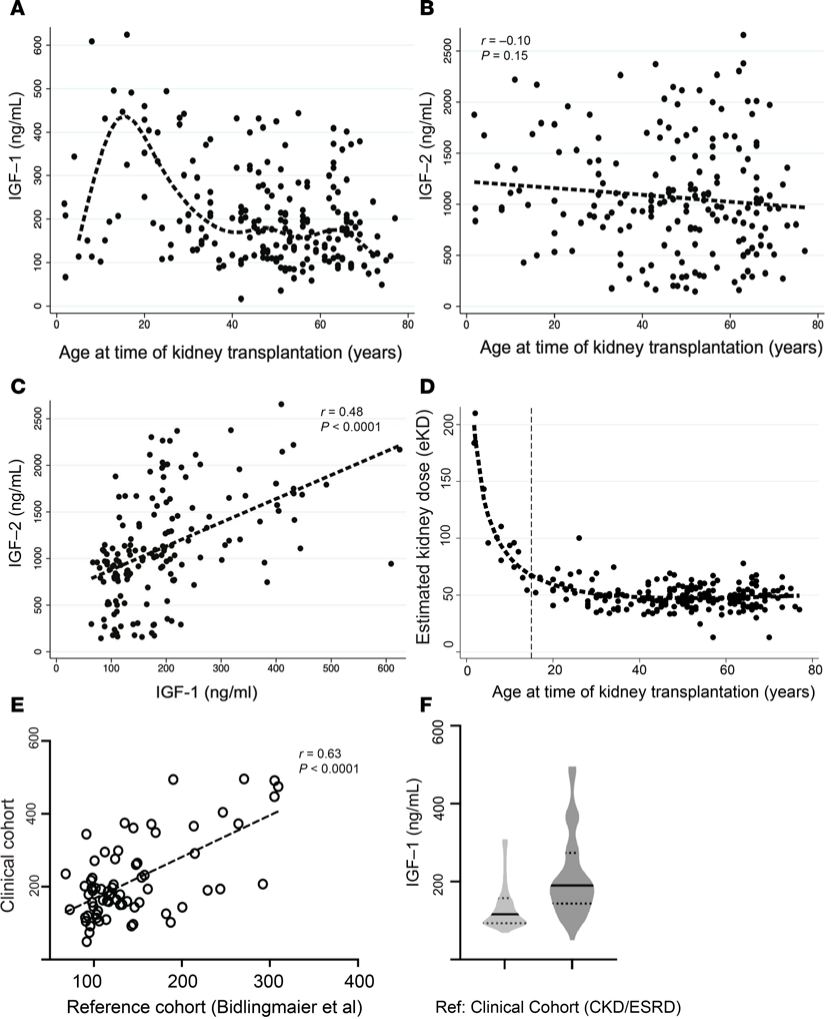
Age-related variation in pretransplant IGF-1/IGF-2 levels and estimated kidney dose (eKD) in kidney transplant recipients. (**A**) Relationship between pretransplant IGF-1 levels and recipient age at KTx. A wide range of IGF-1 levels was observed across all ages, but the average curve is like that of the reference population (Figure 2C). (**B**) Relationship between pretransplant IGF-2 levels and recipient age. A wide range of IGF-2 levels was observed across all ages. (**C**) Pretransplant IGF-1 and IGF-2 levels were correlated (*r* = 0.48, *P* < 0.0001). IGF-2 was below the detection level in 27 samples (12.5%). (**D**) Relationship between estimated kidney dose (eKD, %) and recipient age at KTx. This relationship is similar to that observed in the OPTN dataset (not shown). The eKD is the percentage of normal 2-kidney mass transplanted into each recipient (see Methods). The vertical line denotes age 15 years. (**E**) Correlation plot between measured average IGF-1 levels by age in the clinical cohort of patients with CKD and ESKD before KTx versus average IGF-1 levels by age from Bidlingmaier et al. (30). (**F**) Distribution of average IGF-1 values by age in the healthy reference population compared to the clinical cohort of patients with CKD and ESKD. Although a direct comparison should not be made, as supported by the literature, IGF-1 levels tend to be higher in patients with CKD and ESKD patients (see Discussion).

**Figure 5:**
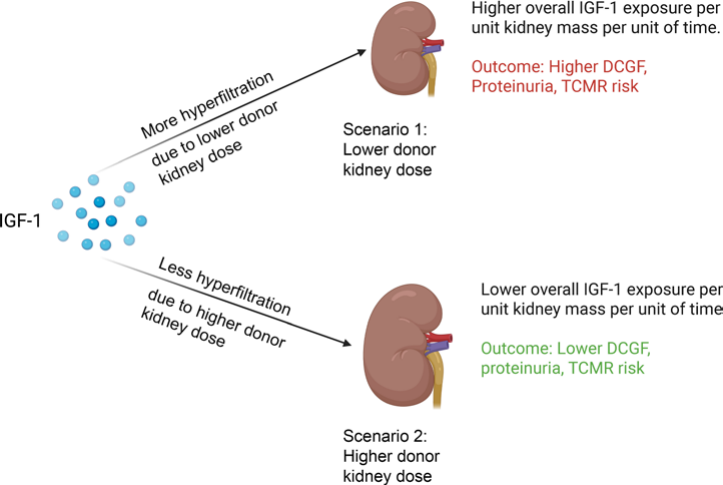
Diagrammatic illustration of how graft IGF-1 exposure is defined by the circulating IGF-1 levels in the recipient at transplantation with the kidney dose that determines the degree of hyperfiltration. At a given IGF-1 level, a lower kidney dose is predicted to be accompanied by increased blood flow and hyperfiltration, which leads to a higher IGF-1 allograft delivery per unit of donor kidney dose per unit of time that in turn is associated with inferior allograft survival. Conversely, at higher kidney doses, hyperfiltration is lower, leading to reduced overall IGF-1 exposure per unit of kidney mass per unit of time and associated with superior allograft survival.

A clinical study of 216 patients was performed, with the sample size determined by a pilot study (see Methods). This study design tested the relationship of early allograft IGF-1 exposure on DCGF as the primary outcome, with high-grade proteinuria (>1 g/g creatinine) and rejection as a secondary outcome. The median follow-up time was 8.6 years. Table 2 details the characteristics of donors and recipients of this cohort. The mean recipient age was 47.4 ± 17.7 (1.8 to 77) years. A single circulating IGF-1 level has shown utility over pulsatile GH levels, given the very low coefficient of variation noted in IGF-1 levels in previous studies (31–34). Serum IGF-1 levels ranged from 16.3 to 625 ng/mL, averaging 207 ± 114 ng/mL. The average serum IGF-2 level was 1068 ± 566 ng/mL, ranging from 144 to 2656 ng/mL. Figure 4A shows IGF-1 levels by age at the time of KTx, emphasizing significant variability but paralleling the population age profile shown in Figure 2, A–C. In contrast, Figure 4B shows no relationship between IGF-2 levels by age at time of KTx, although a significant correlation was observed between IGF-1 and IGF-2 levels (*r* = 0.48, *P* < 0.001) (Figure 4C). Figure 4D shows the relationship between the estimated kidney dose (eKD) delivered at KTx and the age at the time of KTx, where eKD is estimated as the donor-to-recipient BSA ratio multiplied by 0.5 (see Figure 5 and Methods for detailed explanation). Recipients younger than 15–16 years tended to have higher eKD values, reflecting their smaller BSA. Figure 4E depicts the Pearson’s correlation (*r* = 0.63, *P* < 0.0001) between measured IGF-1 levels by age in the clinical study (*y* axis) and population data reported by Bidlingmaier et al. (*x* axis) (30). Figure 4F compares the distribution of measured total serum IGF-1 levels in the reference population versus those in this study’s chronic kidney disease (CKD)/ESRD recipient cohort. Notably, the CKD/ESRD cohort tended to have higher IGF-1 levels than the reference population. This observation is consistent with higher circulating IGF binding protein 3 (IGFBP3) in the CKD/ESRD population, although free IGF-1 levels are reported to be no different between the reference and CKD/ESRD populations (35).

**Figure 6:**
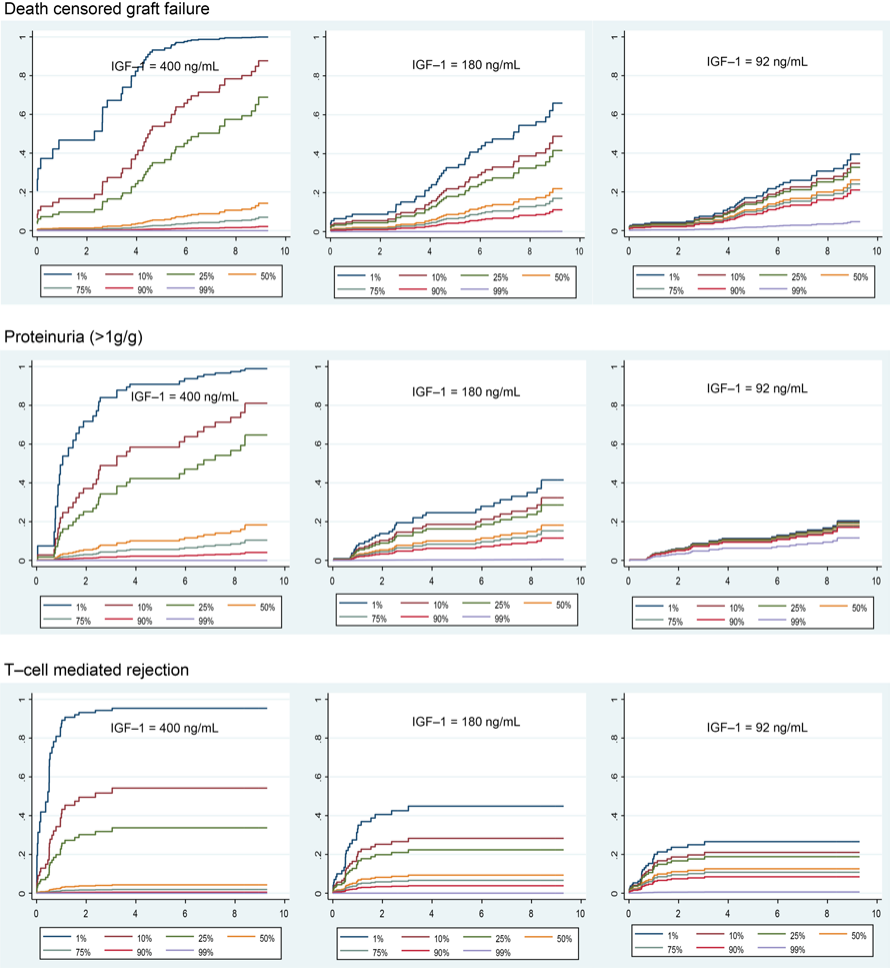
Relationship between IGF-1 levels and outcomes of interest in relation to the estimated kidney dose (eKD) delivered to the recipient at transplantation (*n* = 216). The survival curves to help visualize adjusted outcomes from the multivariable Cox regression model are generated for different IGF-1 values (90th, 50th, and 10th percentiles of the distribution in the clinical cohort) and eKD values (1st, 10th, 25th, 50th, 75th, 90th, and 99th percentiles corresponding to 0.25, 0.37, 0.42, 0.50, 0.56, 0.67, and 1.83 transplanted kidney equivalents). Mean centering was performed for the eKD variable to allow for interpretation of the direct effect of IGF-1 at the mean of eKD in our clinical cohort. For DCGF and proteinuria, the Cox regression models were adjusted for donor age, donor race, donor sex, donor type (living or deceased), donor body surface area, recipient age, recipient race, recipient sex, and cold ischemia time. For the TCMR (and separately for BPAR), adjustments were made for pretransplant calculated panel reactive antibodies class 1 or 2 and the number of previous transplants in addition to the above variables.

Figure 5 provides a schematic representation for how the amount of allograft IGF-1 exposure was defined as the product of the recipient’s circulating IGF-1 levels at time of KTx and the projected degree of hyperfiltration experienced by that graft. The projected degree of hyperfiltration experienced by each graft was defined as the eKD delivered at transplantation. Graft IGF-1 exposure at time of transplantation is therefore given by the pretransplant IGF-1 level multiplied by the eKD (IGF-1 × eKD). Table 3 summarizes the association between allograft IGF-1 exposure (IGF-1 × eKD) with outcomes, including DCGF, proteinuria, and biopsy-proven acute rejection (BPAR), and additional analysis for T cell–mediated rejection (TCMR). To illustrate how these results should be interpreted, the example of DCGF, as shown in Table 3, is used here. The hazard ratio (HR) of the IGF-1 × eKD interaction term is 0.99, indicating that for each 1-unit increase in kidney dose (eKD) (e.g., a 1% increase in kidney dose from 53% to 54%), there is a corresponding 1% decrease in the risk of DCGF. Thus, although the HR value of 0.99 appears small, it is a large effect, as modeled in Figure 6.

#### DCGF.

Forty-four of the 216 patients experienced DCGF by a median of 8.6 years of follow-up. IGF-1 exposure (IGF-1 × eKD) was significantly associated with DCGF (*P* = 0.007) (Table 3). Figure 6 (upper panels) shows the association of graft IGF-1 exposure with DCGF. Higher IGF-1 levels in the setting of smaller kidney dose (thus more hyperfiltration allowing greater graft exposure to the high circulating IGF-1 level) was associated with increased DCGF risk. Conversely, a larger kidney dose (lower hyperfiltration resulting in less graft exposure to circulating IGF-1) was associated with lower DCGF risk even when IGF-1 levels were high. Notably, at lower IGF-1 levels, kidney dose had a reduced effect on DCGF risk. For the complete multivariable Cox models, refer to Supplemental Table 1; supplemental material available online with this article; https://doi.org/10.1172/jci.insight.188485DS1 Additional sensitivity analyses are shown, including adjustment of pretransplant sensitization (Supplemental Table 2), development of posttransplant de novo donor-specific antibodies (dnDSAs) (Supplemental Table 3), and BPAR (Supplemental Table 4). In all sensitivity analyses, graft IGF-1 exposure continued to be significantly associated with increased DCGF, suggesting a strong independent effect of IGF-1 exposure on long-term graft survival.

#### Proteinuria (>1 g/g creatinine in urine).

Proteinuria is a well-established surrogate for long-term allograft outcomes (36). Thirty-three of the 216 patients developed proteinuria by a median of 2.4 years after KTx. As noted above for DCGF, graft IGF-1 exposure (IGF-1 × eKD interaction) was significantly associated with proteinuria (*P* < 0.01) (Table 2). Figure 6 (middle panels) shows the effect of IGF-1 exposure on proteinuria. Like DCGF, higher graft IGF-1 exposure was associated with increased proteinuria, while this effect was less pronounced at lower IGF-1 exposure. For the complete multivariable Cox models, see Supplemental Table 5.

#### BPAR.

Forty-four patients were identified as experiencing BPAR from routine surveillance biopsies at 3, 6, and 12 months and clinically indicated biopsies. The median time to any rejection was 0.95 years, with the median times for TCMR at 0.54 years, antibody-mediated rejection at 2.9 years, and mixed rejection at 3.1 years. In the sensitivity analysis, higher graft IGF-1 exposure (IGF-1 × eKD) was associated with an increased tendency toward BPAR (*P* = 0.07) and a significant association with TCMR (*P* = 0.04) (Table 2). Figure 6 (bottom panels) shows the effect of graft IGF-1 exposure on TCMR risk. For the complete multivariable Cox models, see Supplemental Tables 6 and 7.

Since donor and recipient races had large effect sizes and are independently associated with DCGF, proteinuria, and TCMR, we conducted an additional sensitivity analysis to evaluate whether the effect of graft IGF-1 exposure on outcomes might be modulated by the recipient or donor race. In this analysis, the interaction term between IGF-1 and recipient or donor race was not significant for any of the evaluated outcomes (Supplemental Table 8, A–F), consistent with the impact of graft IGF-1 exposure on DCGF, proteinuria, and TCMR being independent of race.

Supplemental Table 9A shows the clinically designated causes of graft loss in the cohort, with chronic allograft nephropathy (CAN)/chronic rejection being the leading cause assigned for graft loss for which diagnostic biopsies were performed.

Supplemental Figure 2, A–C shows the Kaplan-Meier curves for DCGF, proteinuria, and TCMR for all patients in this cohort.

#### Relationship of circulating IGF-2 level to allograft outcome.

No significant relationship was detected between pretransplant IGF-2 levels and DCGF, proteinuria, or alloimmune responses (data not shown).

### Cohort 3: genotype study

**Figure 7:**
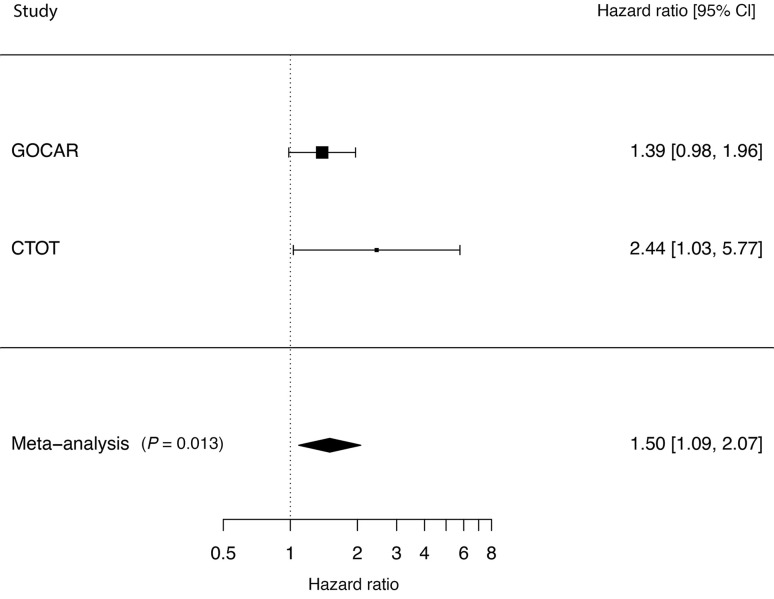
Meta-analysis using the genomics of chronic allograft rejection (GoCAR) and the clinical trials in organ transplantation (CTOT) studies. In the GoCAR study, the risk of DCGF, with an adjusted hazards ratio (aHR) of 1.38, did not reach clinical significance (P = 0.06), while in the CTOT study, the aHR was 2.44, which achieved statistical significance (P = 0.001). The meta-analysis included 527 KTx recipients from the GoCAR study and 197 patients from the CTOT study and was significant (P = 0.013). There was a 50% increase in the risk of DCGF associated with recipient carriage of a single rs35767 risk allele (aHR = 1.50, P = 0.01).

Genetic factors are reported to account for 38% of the variance in IGF-1 levels (37). The SNP variant (*rs35767*) in the regulatory region of the *IGF1* gene is associated with elevated circulating IGF-1 levels (38–41). Thus, if our hypothesis is correct, a genetic variant in the *IGF1* gene regulatory region that increases the levels of circulating IGF-1 in the recipient would also be expected to be associated with inferior long-term KTx outcomes. A multivariable analysis of the NIH-funded Genomics of Chronic Allograft Rejection (GoCAR) (*n* = 527) and Clinical Trials in Organ Transplantation (CTOT) (*n* = 197) cohorts, supplemented by a meta-analysis pooling these studies (*n* = 724) is depicted in Figure 7 (42–45). These data suggest that possessing even just 1 risk allele is associated with a 50% (HR = 1.50, *P* = 0.01) increase in the DCGF risk. Table 4 shows the multivariable analysis to explore the effect of risk allele *rs35767* on recipient DCGF risk. The clinically designated causes of graft loss in the GoCAR and the CTOT cohorts are shown in Supplemental Table 9, B and C. These data further support the hypothesized relationship between IGF-1 exposure and DCGF.

### Cohort 4: analysis of IGF-1 pathway transcripts in healthy and transplanted kidneys

**Figure 8:**
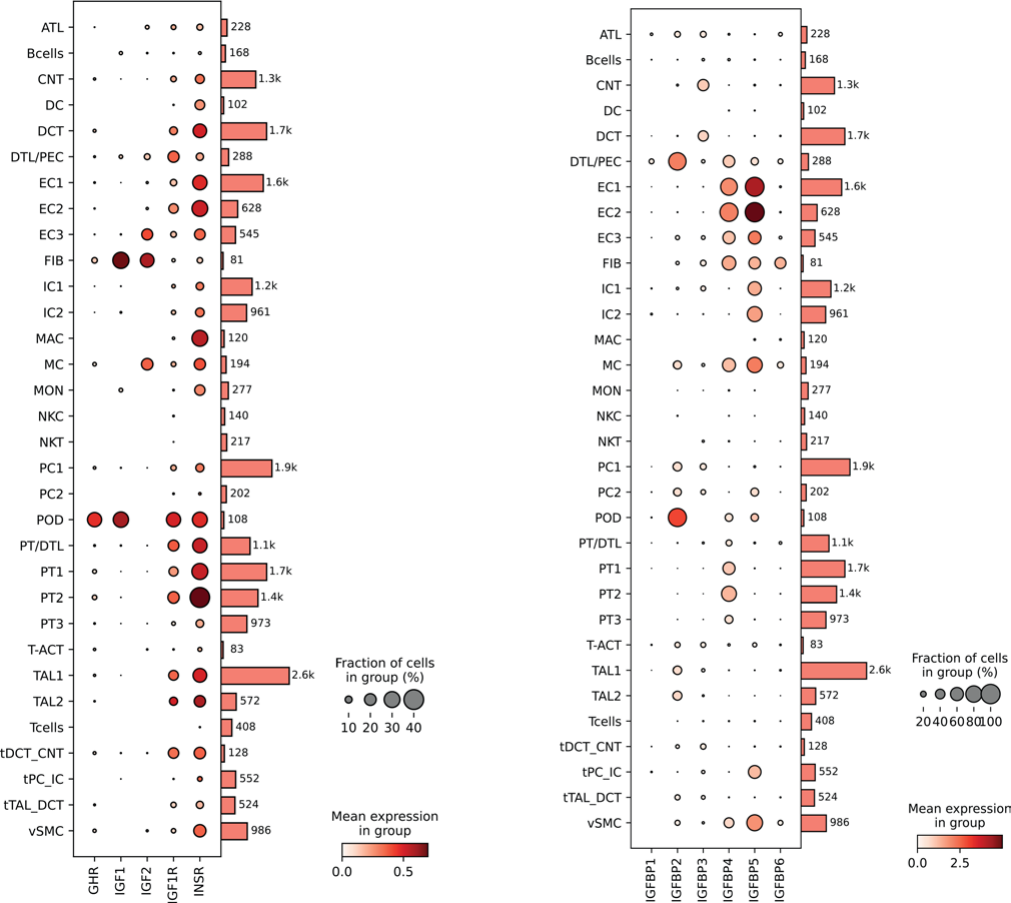
Single-cell transcriptomic analysis of IGF pathway transcripts in kidney cells derived from normal kidney biopsies obtained before transplantation. Data are sourced from a previously reported dataset, wherein the criteria for cell identification are detailed (46). The number of cells available for analysis for each cell type is displayed to the right of each panel. The cell types are listed alphabetically from top to bottom (definitions below). For each transcript, the dot size represents the proportion of cells within the cell type that expressed detectable transcripts, while the dot color’s intensity reflects the average amount of transcript detected across all cells. The dot size and intensity calibration are shown at the lower right of each panel. The panels display transcripts for insulin-like growth factor 1 (*IGF1*), IGF-1 receptor (*IGF1R*), growth hormone receptor (*GHR*), insulin-like growth factor-2 (IGF2), insulin receptor (*INSR*), and IGF binding proteins 1–6 (IGFBP1–6). Cell designations are as follows: ATL, ascending thin limb; BCells, B cells; CNT, connecting tubule; DC, dendritic cells; DCT, distal convoluted tubule; DTL, descending thin limb; EC1, arteriolar endothelial cells; EC2, glomerular endothelial cells; EC3, peritubular capillaries; FIB, fibroblasts; IC1/2, intercalated cells 1 and 2; MAC, macrophages; MC, mesangial cells; MON, monocytes; NKC, natural killer cells; NKT, natural killer T cells; PC1/2, principal cells cluster 1 and 2; POD, podocytes; PT/DTL, proximal tubule/descending thin limb; PT1/2/3, proximal tubule subclusters 1, 2, and 3; T-ACT, activated T cells; TAL1/2, thick ascending limb subcluster 1 or 2; TCells, T cells; tDCT_CNT, transitional cells of DCT with CNT; tPC_IC, transitional principal and intercalated cells; tDAL_DCT, transitional cells between TAL and DCT; vSMC, vascular smooth muscle cells.

Single-cell RNA-seq was used to analyze preimplantation healthy donor biopsies compared with surveillance biopsies from first-year allografts in which no pathologic abnormality was detected (46). In normal kidneys, *IGF1* transcripts were predominantly expressed in podocytes and fibroblasts, with other kidney cell types showing lower-level expression (Figure 8). Podocytes highly expressed GH receptor (*GHR*) transcripts. *GH* transcripts were not detected in any kidney cell type (data not shown). *IGF1R* and insulin receptor (*INSR*) transcripts were broadly expressed, particularly in podocytes and specific proximal tubular cells. These transcripts tended to be expressed similarly, reflecting that they are gene reduplicates and form hybrid receptors responsive to IGF-1 (47). Expression of transcripts for *IGFBP1–6* tended to be cell specific, suggesting a refined regulatory mechanism within specific renal cell types (48).

**Figure 9:**
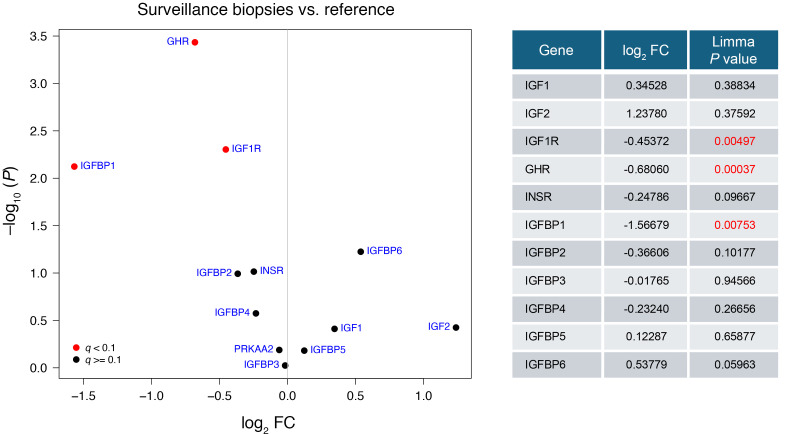
Volcano plot depicting the differential expression analysis of selected genes within the IGF-1 signaling pathway using pseudobulked single cell RNA-seq. This analysis contrasts gene expression in first-year kidney allografts with that in binephric healthy individuals. In the plot, individual genes are represented by points, plotted according to their fold change (*x* axis) and the negative logarithm (base 10) of their *P* value (*y* axis). Points colored red denote genes with FDR-adjusted *P* values (using the Benjimini-Hochberg method) below a threshold of 0.1. In this analysis, both *GHR* and *IGF1R* transcripts were significantly downregulated in allografts. In contrast, *IGF1* transcripts were not significantly downregulated.

Pseudobulk transcript analysis was used for comparison of first-year allograft to overcome the recently recognized statistical limitations and false discovery rates inherent in single-cell analysis, particularly where recovered cell numbers of certain cell types are limited (e.g., podocytes) (49). *GHR* and *IGF1R* transcript expression was significantly reduced in allografts, mirroring reports from rodent UniNx models (Figure 9) (28, 50). Notably, *IGF1* transcripts were not increased in first-year grafts. These data support the hypothesis that the allograft’s normal compensatory adaptive response is to downregulate its intrinsic *GHR* and *IGF1R* in response to increased exposure to extrinsic GH and IGF-1 from the circulation because of hyperfiltration.

## Discussion

The data shown from 4 independent studies support the hypothesis that KTx longevity is related to IGF-1 exposure from the recipient’s circulation that occurs early after KTx. A population-level study identified that patients undergoing KTx in adolescence and early adulthood, when ambient GH and IGF-1 levels are highest, have the highest intermediate and long-term DCGF rates, while lower IGF-1 levels at extremes of age were associated with lower DCGF rates. This translated to a major difference in median allograft longevity from about 12 years for a 15-year-old recipient versus 20 years for a 60-year-old recipient. Higher graft IGF-1 exposure at time of transplantation was associated with a higher risk of DCGF (primary outcome) and proteinuria (secondary outcome). Conversely, lower IGF-1 exposure was associated with a lower risk for DCGF and proteinuria. Notably, while not powered for immune-mediated effects, our analysis revealed an increased risk of TCMR associated with higher graft IGF-1 exposure and vice versa. The association between allograft IGF-1 exposure and outcomes was further buttressed by genotype studies using 3 NIAID KTx cohorts wherein an *IGF1* SNP linked to high circulating IGF-1 levels was associated with a higher DCGF risk (38–40). Finally, in alignment with prior UniNx studies, transcriptomic data showed no evidence for increased intrinsic kidney *IGF1* transcripts in first-year allografts. Furthermore, intrinsic kidney *GHR* and *IGF1R* transcripts were downregulated in allografts, suggesting the presence of an inhibitory feedback loop to minimize the effects of excess graft exposure to extrinsic GH and IGF-1 from the circulation because of hyperfiltration (28, 50). Together, these studies strongly support a link between GH/IGF-1 axis–mediated signaling and kidney allograft failure.

The relationship of circulating IGF-1 level to DCGF risk depends on the kidney dose delivered at KTx, which likely determines relative kidney blood flow and degree of hyperfiltration (8). Higher circulating IGF-1 levels and a smaller kidney dose (predicted to cause higher graft IGF-1 exposure) were associated with higher DCGF, proteinuria, and TCMR risk. Conversely, at low circulating IGF-1 levels, the donor kidney dose is less consequential for graft outcomes, likely due to lower IGF-1 exposure regardless of the delivered kidney dose. This observation has potential implications for expanding the deceased donor kidney pool, as discussed below.

Podocytes express high levels of *GHR*, *IGF1R*, and *IGF1* transcripts, implying that the kidney also has an intrinsic podocyte-based IGF-1 regulatory mechanism, a finding consistent with the kidney being a net producer of IGF-1 (51, 52). High GH exposure from hyperfiltration at the time of KTx would tend to activate podocyte GHRs to drive podocyte production of IGF-1, and the combined effects of GH and IGF-1 (acting through the podocyte IGF-1R that is also expressed at high levels by podocytes) would promote mTORC1 activation to drive podocyte hypertrophy. We conjecture that the observed reduction in *GHR* and *IGF1R* transcript expression in allografts with no detectable pathologic abnormality reflects a broader adaptive response to protect against excess GH and IGF-1 exposure in hyperfiltering allografts. Similar findings of reduced *IGF1R* transcript expression in hyperfiltering adult rodent remnant kidney models have previously been reported (50). Thus, the net effects of hyperfiltered GH (that would tend to increase podocyte IGF-1 production) versus high allograft exposure to hyperfiltered IGF-1 from blood (that hypothetically would tend to downregulate intrinsic *IGF1R* transcript expression) could balance out in the stable allograft, resulting in no significant change in the IGF-1 transcript level, as observed in our data.

IGF-1 exposure was also associated with a trend toward increased TCMR Banff grade 1A or higher (53). This observation is consistent with a rodent model of allotransplantation, where smaller kidney doses exhibited higher rejection rates and CAN (54, 55). Early reintroduction of additional kidney dose was reported to reverse rejection and mitigate CAN, consistent with kidney dose being related to rejection and overall graft survival. The reduction in rejection associated with a larger kidney dose is consistent with the hypothesis that increasing the donor kidney dose would reduce hyperfiltration-induced allograft IGF-1 exposure, potentially diminishing alloimmune events and other processes that drive rejection.

A single *rs35767* SNP risk allele was associated with a 50% elevation in risk of DCGF. This variant’s minor allele frequency (MAF), as reported in the ALFA project, was 0.18 for adenine (56). The highest MAF is in African populations and Americans of immediate African ancestry (MAF = 0.48), with the lowest frequency observed in individuals of European ancestry (MAF = 0.16). Similar MAF was observed across self-reported ancestry in a CKD cohort (NEPTUNE) (Supplemental Table 10). Corroborating our data, a recent report from Japan has also linked this genetic variant with worsening kidney function over the long term (38). Further studies are needed to understand the contributions of SNP *rs35767* to both CKD prevalence and worse allograft survival, particularly among the African American community in the United States (57, 58).

Interpreting the role of IGF-1 in the allograft requires understanding the GH/IGF-1 axis biology in the kidney (51, 59, 60). The liver releases IGF-1 under the control of pituitary-derived GH. The kidney is also a net producer of IGF-1, with single-cell analysis demonstrating that podocytes and fibroblasts are major cell types expressing *IGF1* transcripts (Figure 8) (52). In circulation, free IGF-1 makes up only about 1% of total IGF-1 in the blood. The remaining 99% of IGF-1 (and IGF-2) circulates bound to IGFBP1–6, a family of gene-reduplicated proteins that bind IGF-1 with 10- to 100-fold higher affinity than the IGF-1R, thereby regulating free IGF-1 availability (61). IGFBPs release IGF-1 locally in response to proteolytic and stereochemical modulations induced by binding to matrix and other molecules (61–63). The major IGFBPs in the blood (IGFBP3 and IGFBP5) also bind to acid-labile subunit (ALS) in blood to form trimolecular 150-kDa complexes that are too large to be filtered by the normal glomerulus and have a longer half-life (64, 65). Both free IGF-1 (7.5 kDa) and the IGF-IGFBP bimolecular (25–45 kDa) complexes would be expected to cross the normal glomerular filtration barrier to gain access to the urinary space, especially under hyperfiltration conditions. Increased renal blood flow would also increase IGF access to the kidney interstitial space and the basolateral surface of tubular cells via peritubular capillaries (66, 67).

GH is released in a pulsatile and diurnal manner, making random measurements of limited use. Thus, a single IGF-1 measurement is routinely used as a readout for net GH effects, given the low coefficient of variation in IGF-1 measurements (32–34, 68). IGF-1 is released in response to GH acting on the GHR in the liver and other cells that express the GHR, including podocytes. GH and IGF-1 in combination can therefore both act via mTORC1 to amplify cell hypertrophy and hypertrophic stress. This is illustrated by murine models where overexpression of IGF-1 causes glomerular enlargement but not glomerulosclerosis, while overexpression of GH also leads to increased IGF-1, causing both glomerular enlargement and glomerulosclerosis (69). In this report, we use the term “GH/IGF-1 axis” to emphasize that the effects associated with IGF-1 may also be caused in part by GH.

Advanced CKD and ESRD are accompanied by increased circulating GH levels, although free IGF-1 levels are in the normal range (35, 70–72). IGFBP3, the major IGFBP in blood, is increased in CKD, thereby accounting for the increase in total IGF-1 level (bound plus free IGF-1) that was also observed in this study (Figure 4F) (35). ALS levels remain in the normal range in CKD and IGF-1 production, and its metabolic clearance rate is comparable in CKD and healthy individuals (35). The total IGF-1 levels in the clinical study varied with recipient age in parallel to those reported in the reference population (Figure 4A), although the levels were slightly higher (Figure 4, E and F), as expected if IGFBP3 levels are higher in CKD/ESKD. We expect that GH and IGFBP3 levels will revert to normal values for age after improved kidney function after transplantation.

IGF-2 levels in blood are generally 3- to 5-fold higher than IGF-1 levels (59). IGF-2 has comparable binding to IGFBPs and ALS and, like IGF-1, activates the IGF-1R to promote growth (20–22). Therefore, higher pretransplant IGF-2 levels might also have been expected to be related to allograft longevity. However, we observed no statistically significant relationship between IGF-2 alone or its interaction with kidney dose with any tested outcomes. Notably, in our rodent UniNx model, IGF-1 was preferentially hyperfiltered to appear in the urine compared with IGF-2, possibly due to differential binding to its carrier IGFBPs and ALS, making it less hyperfilterable (27).

Medication non-compliance has been suggested as a cause of increased graft loss in teenagers (73, 74). Our population-level analysis demonstrated that those who were teenagers age at time of KTx were not associated with increased graft loss by 1 year after KTx. This contrasts with intermediately after (3 years) and especially long-term (10 years) after KTx, where there was a marked increase in graft loss in age groups with higher IGF-1 levels (adolescence and young adulthood). The relative contributions of IGF-1 and non-compliance to shorten graft survival will need to be evaluated by a dedicated study.

A clinical question arising from this work is why the single remaining kidney in the living kidney donor does not experience higher kidney failure risk due to the GH/IGF-1 axis effect. This might arise from the donor having a 50% kidney dose remaining, which is predicted to impose a relatively small risk by 10 years of follow-up (Figure 6, top panels). Other potential mitigating factors include the fact that the average live kidney donor is 40 years of age when IGF-1 levels are not at their highest and fall over time, an absence of an alloimmune milieu, lower GH levels in live donors (versus CKD and ESKD which are high-GH states) (70–72), and the presence of innervation that may protect against hyperfiltration. Regardless, the current data do suggest that there is indeed a relative increase in donor risk of ESKD in live kidney donors observable by 15 years after nephrectomy, although this absolute risk is relatively small (75). However, concern remains that the risk of CKD and ESKD will increase as longer-term data are accumulated (76). These observations point to the need for careful donor follow-up and mitigation of compounding factors such as hypertension, obesity, and diabetes, as well as IGF-1 levels that may amplify the risk for nephron loss in kidney donors (77). A prediction from our clinical cohort data is that increasing the transplanted kidney dose will minimize the impact of high IGF-1 levels and thus improve allograft outcomes, particularly for adolescents and younger adults. Conversely, in older recipients with lower IGF-1 levels, a smaller kidney dose, including kidneys with some glomerulosclerosis currently discarded (78, 79), would be expected to have good long-term survival. A second prediction is that reducing early allograft exposure to GH/IGF-1 will improve long-term allograft survival. Angiotensin-converting enzyme (ACE) inhibition, which reduces hyperfiltration, will limit graft exposure to GH/IGF-1, would be expected to be protective. Several ACE inhibitor trials in KTx have shown little clinical benefit (80–82). However, recent evidence suggests that very early versus later drug initiation may lessen tissue injury and improve long-term function surrogates (83). Our animal model and clinical data also suggest that the lack of an observed ACE inhibition protective effect could be because the drug was started well after KTx due to concerns of hyperkalemia, reduced renal perfusion, hypotension, and anemia (84). Modeling data suggest that significant growth events that ultimately determine allograft destiny can occur very early after transplantation, and thus, initiating ACE inhibitors outside this window minimizes potential benefits (16, 27). Specific IGF-1R blockade (currently in use to treat immune-mediated thyroid eye disease) and/or targeting GH (by GH-releasing hormone [GHRH] inhibition using octreotide or its analogs) may be feasible to enhance allograft longevity (85).

Unequivocal proof that the GH/IGF-1 axis drives shorter graft survival will require prospective clinical trials that specifically limit allograft exposure to IGF-1. Another limitation of this report is that kidney dose was extrapolated through the donor-to-recipient BSA ratio as a surrogate for its projected hyperfiltration effect, although the use of this surrogate is supported by the strong correlation between BSA and kidney size (86–88). Additionally, the lack of detailed genetic information precluded testing for whether the effect of IGF-1 on outcomes was conditional on high-risk APOL1 risk alleles. Importantly, in this report, we use “hard” outcomes (DCGF, proteinuria, and protocol biopsy analysis) for comparison to graft IGF-1 exposure. Clinically designated “causes” of graft failure for each cohort are also provided (Supplemental Table 9, A–C).

**Figure 10:**
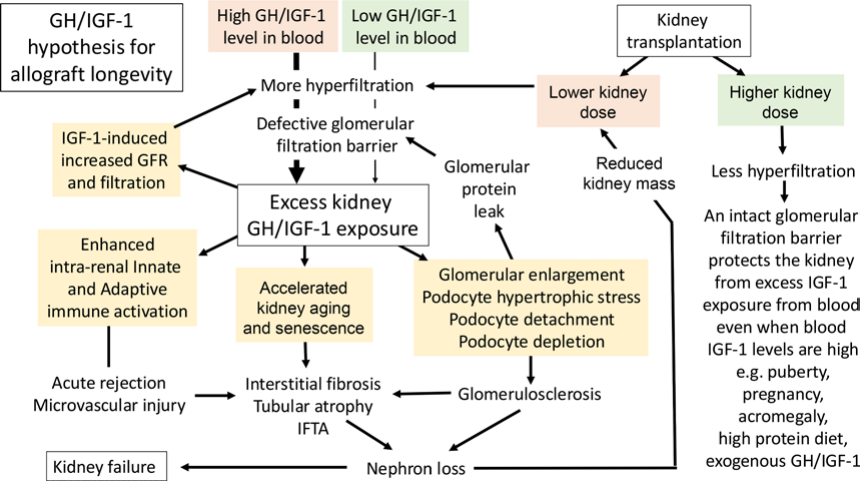
Diagrammatic illustration of the allograft IGF-1 exposure hypothesis for shorter KTx survival. The single kidney state sets the allograft up for increased IGF-1 exposure dependent on the donor kidney dose and, thereby, the extent of hyperfiltration. A higher IGF-1 exposure, with its potential diverse downstream consequences, is shown in yellow boxes. We hypothesize that in addition to its role in early glomerular hypertrophy and podocyte stress (11–13, 16), IGF-1 is likely to amplify the glomerular filtration rate by a nitric oxide–mediated vasodilatory response (3), with higher IGF-1 levels leading to a higher vasodilatory response. These data are also supported by the observations that an IGF-1R and nitric oxide inhibitor (3, 92, 93) can mitigate hyperfiltration. Furthermore, IGF-1 could promote fibroblast activation in KTx as it does in other model systems (94, 95), leading to the development of CAN purported to be a final common pathway of graft loss (96). In addition, IGF-1 is known to enhance immune activation, which was also supported in our analysis, where an increased risk of TCMR was observed. The insulin–insulin-like growth factor system (IIS) drives shorter lifespans in worms, flies, and mice. We speculate that similar IGF-1 signaling via IGF-1R may also accelerate senescence, which is reported in kidney allografts. As noted by our analysis, larger kidney doses reduce kidney exposure to hyperfiltered IGF-1, thereby relatively protecting the normal 2-kidney state from the downstream effects of high circulating IGF-1 levels under different conditions (e.g., during puberty and diseases associated with high IGF-1 levels, including acromegaly, pregnancy, and exogenous GH and IGF-1 treatments).

In summary, we highlight an association between circulating IGF-1 levels at the time of KTx and long-term allograft survival. Figure 10 summarizes putative mechanisms for how increased graft exposure to GH/IGF-1 axis pathways can result in diverse pathologic processes constituting CAN that is associated with shorter kidney lifespan.

## Methods

### Sex as a biological variable

Both sexes were included in this study as we expected the findings of this study to be similar across to both sexes.

### Cohort 1: population-level study

Data from the OPTN database (October 1, 1987, to March 31, 2022) were analyzed. After applying exclusion criteria (Supplemental Figure 1), 366,404 observations remained for analysis. The incident DCGF rate for recipients ages 0–60 years was determined using the Kaplan-Meier estimator, censoring for death, retransplantation, loss to follow-up, or the end of the follow-up period. The “stptime” command was used to generate incident DCGF rates per 1000 patient-years by age at KTx and age at time of DCGF. The assumption of Cox’s proportional hazards was verified using Schoenfeld residuals, with the Stata command “estat phtest” employed for this purpose. The incident DCGF rate was also parsed by time after KTx to generate incident DCGF rates for 1-year, 3-year, and 10-year outcomes. Furthermore, we obtained the time by which 50% of allografts are lost, censoring for death for each age group to obtain a “median allograft survival” by age. The IGF-1 level for each age group for males and females was obtained from population-level average serum IGF-1 levels by age and sex across 0–60 years from Bidlingmaier et al. (*n* = 15,014) (30). A Welch’s *t* test assuming unequal variances for males and females was used to assess the overall difference in mean IGF-1 levels between males and females and was not statistically significant.

### Cohort 2: clinical study

Serum samples stored before KTx were analyzed for 216 consecutive recipients who underwent KTx at this institution starting July 1, 2013. A Luminex assay was utilized to measure total IGF-1 and IGF-2 levels using MILLIPLEX MAP human IGF-I and IGF-II magnetic bead panels (EMD Millipore) in duplicate.

#### Power analysis.

A sample size estimation of 211 patients was calculated using an HR of 2.0, an event probability of 0.31, and a power of 0.8. The event probability was based on an interim analysis of the initial 106 recipients. The HR of 2.0 used for analysis is a conservative effect size compared with the HRs from previously reported UniNx animal data, which ranged from 4- to 8-fold (4, 5, 27).

### eKD

An eKD was calculated for each recipient using the donor-to-recipient BSA ratio to modify the value of 50% of the normal kidney dose that is routinely provided by a single KTx, and that replaces the function normally provided by 2 kidneys (100%). For example, if a donor and recipient have the same BSA, the ratio would be 1, which equates to 50% of the ideal 2-kidney complement. A donor BSA greater than the recipient BSA means that more than 50% eKD has been delivered (greater than 1-kidney complement), and if the donor BSA is less than the recipient BSA, then the eKD delivered will be less than 50% (less than 1-kidney complement).

#### Allograft IGF-1 exposure.

Allograft IGF-1 exposure is a function of the IGF-1 concentration in the recipient’s blood at time of transplantation multiplied by the eKD delivered. Donor-to-recipient BSA has long been used as a surrogate for the amount of hyperfiltration, and eKD is a function of this measure (8). Thus, we modeled allograft IGF-1 exposure at KTx by the IGF-1 level and its interaction with the kidney dose (IGF-1 × eKD). Figure 5 provides a visual representation of how allograft IGF-1 exposure was modeled. Allograft IGF-2 exposure was similarly modeled.

### Outcomes of interest

The primary endpoint of this study was the time to DCGF from any cause. Secondary endpoints included the development of proteinuria (defined as ≥1 g/g of urinary creatinine) and the occurrence of BPAR. Sensitivity analyses were performed to determine whether the effects of IGF-1 exposure on DCGF were independent of post-KTx complications such as BPAR (60), the emergence of de novo dnDSAs, or predictors of pretransplant allosensitization, including the number of previous transplants and calculated panel reactive antibodies (cPRAs) for HLA class I or II. Additional sensitivity analyses were performed on the BPAR subgroup, specifically TCMR (53). Due to the low number of events in the antibody-mediated rejection and mixed rejection categories, no additional sensitivity analysis was performed.

#### Survival analysis.

We utilized multivariable Cox regression models to analyze the effect of IGF-1 exposure (IGF-1 × eKD interaction) on primary and secondary study endpoints. These models were adjusted for known covariates influencing long-term outcomes, which were determined a priori.

Continuous variables are presented as mean ± SD, and categorical variables are reported as proportions. The assumption of Cox’s proportional hazards was verified using Schoenfeld residuals, with the Stata command “estat phtest” employed for this purpose. After multivariable Cox regression, the “stcurve” command was utilized to visualize covariate-adjusted outcomes across different IGF-1 values (90th, 50th, and 10th percentile distribution) and eKD values (at the 1st, 10th, 25th, 50th, 75th, 90th, and 99th percentiles). The eKD was mean-centered to allow the interpretation of the independent IGF-1 effect on the outcomes of interest at the mean eKD in this cohort. Nonlinear relationships were evaluated using quadratic, cubic, and fractional polynomial regression. The model that visually fitted the data best was used and confirmed by the likelihood ratio test and the model’s *R*^2^ values. All analyses used Stata/MP 17.0 and GraphPad Prism version 10.4.1.

### Cohort 3: genotype studies

Genotype-to-phenotype associations were explored through a meta-analysis of 3 published genotype/phenotype studies focused on KTx outcomes: Genomics in Chronic Allograft Rejection (GoCAR), and the Clinical Trials in Organ Transplantation 01 (CTOT01) and CTOT17 (44, 89, 90). The SNPs from IGF-1 were associated with DCGF in GoCAR and CTOT01 and CTOT17 using multivariable Cox regression by donor age, recipient body weight, recipient age, and HLA mismatch score. A fixed-effect meta-analysis with results from each cohort was conducted with R package “metafor.” Note that due to the unavailability of donor weight data or BSA, the models included recipient weight as a variable to account for the recipient’s body size.

### Cohort 4: transcriptomic analysis of IGF-1 signaling in healthy and transplanted kidneys

#### Dataset.

Reference healthy human transcriptomic data from the Human Kidney and Transplant Transcriptome Atlas (HKTTA) were utilized to define the transcriptional landscape of the IGF-1 signaling pathway in a healthy binephric state (46, 91). See Supplemental Methods for details of single-cell and pseudobulk analyses.

### Study approval

The Institutional Review Board at the University of Michigan (IRB no. HUM00228292) approved this study.

### Data availability

The OPTN data are publicly available upon request from OPTN. All other deidentified clinical data can be obtained by request from the corresponding author. Values for all data points in graphs are reported in the Supporting Data Values file.

## Author contributions

RCW and ASN planned the concepts and studies jointly over more than 8 years, building on RCW’s podocyte depletion hypothesis as it applied to the single kidney state and kidney transplantation. ASN and RCW jointly wrote drafts of the manuscript. MC identified and pulled samples for analysis, performed IGF-1 and IGF-2 assays, and provided critical feedback regarding sample quality control and planning of the individual patient study. All data analysis was performed by ASN, VN, DF, RM, and JH and interpreted by ASN, RCW, JAB, VN, PH, WZ, MCM, MK, EO, and MC. CTOT and GoCAR data were provided by PH, WZ, MCM, ZS, and ZZ. MCM, SN, ND, and KS collated all the clinical data. EO was responsible for single-cell preparation in the initial study by Menon et al., which provided the single-cell transcriptomic data. All authors agree to the final version of this manuscript and consented to submission.

## Supplementary Material

Supplemental data

ICMJE disclosure forms

Supporting data values

## Figures and Tables

**Table 1:**
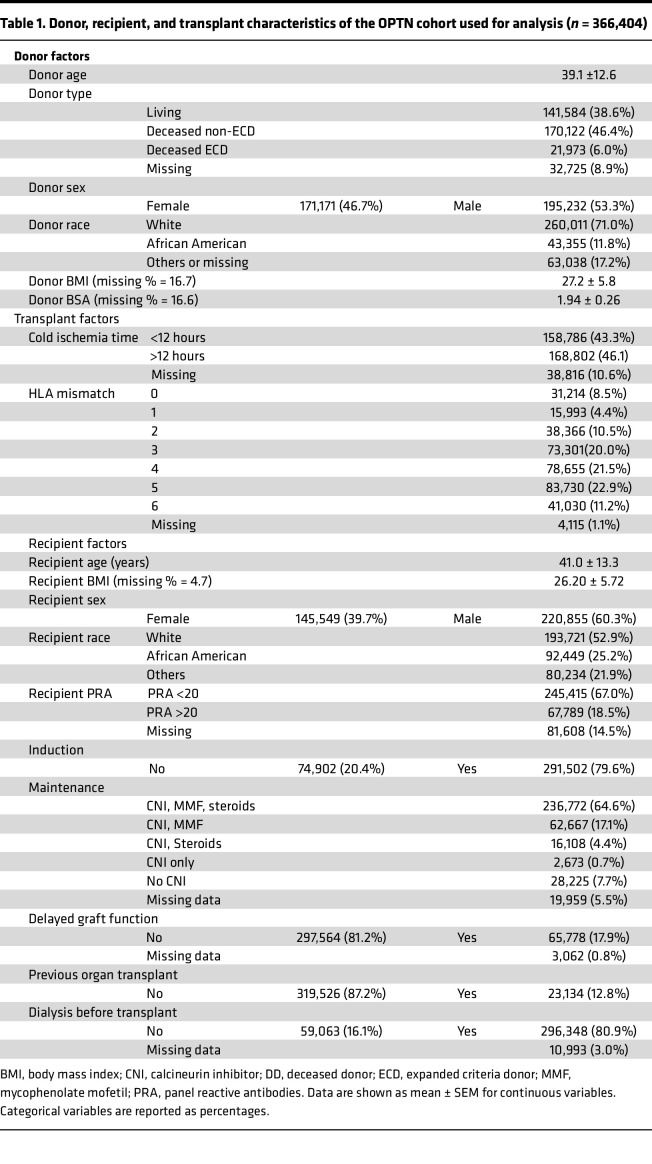
Donor, recipient, and transplant characteristics of the OPTN cohort used for analysis (*n* = 366,404)

**Table 2:**
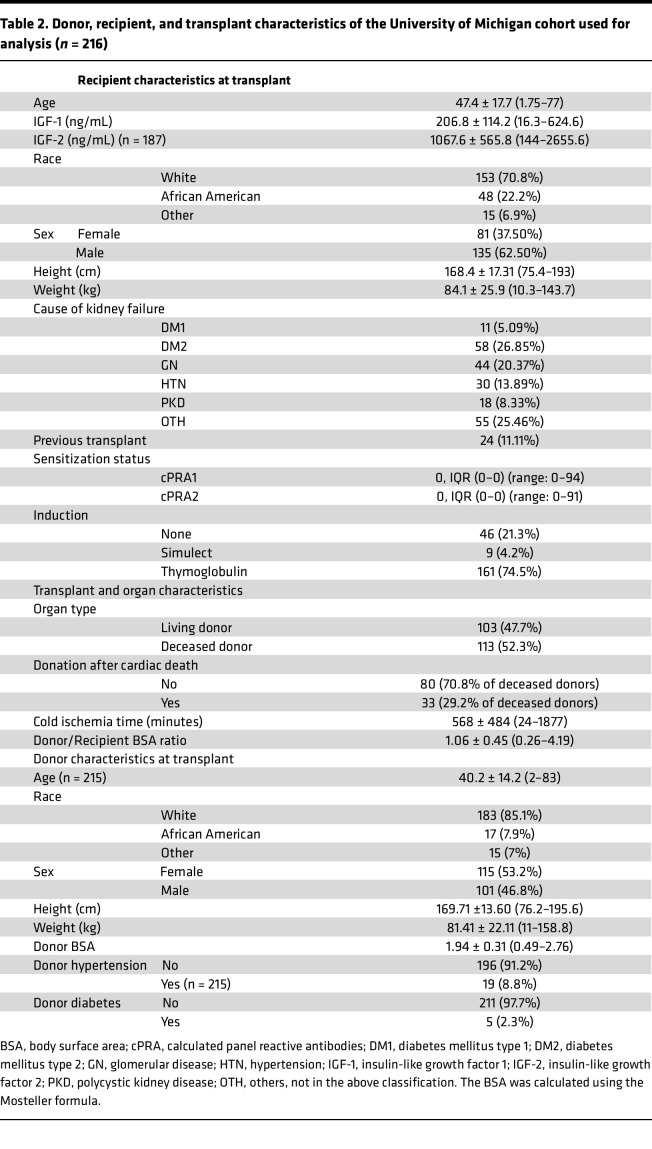
Donor, recipient, and transplant characteristics of the University of Michigan cohort used for analysis (*n* = 216)

**Table 3:**
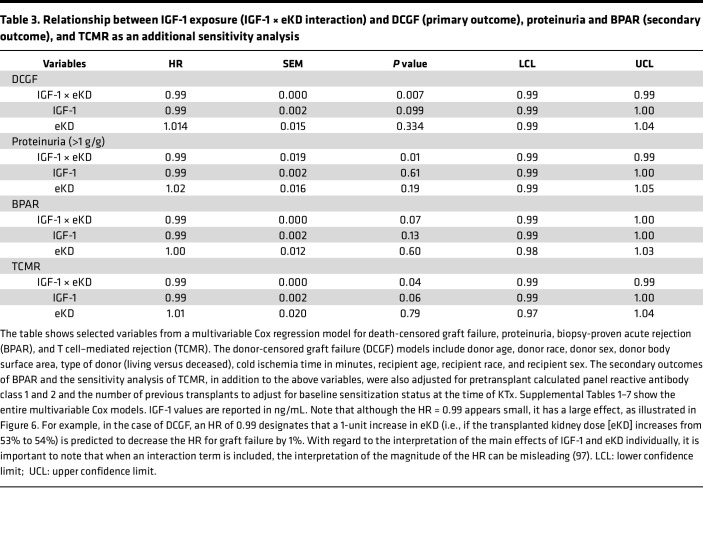
Relationship between IGF-1 exposure (IGF-1 × eKD interaction) and DCGF (primary outcome), proteinuria and BPAR (secondary outcome), and TCMR as an additional sensitivity analysis

**Table 4:**
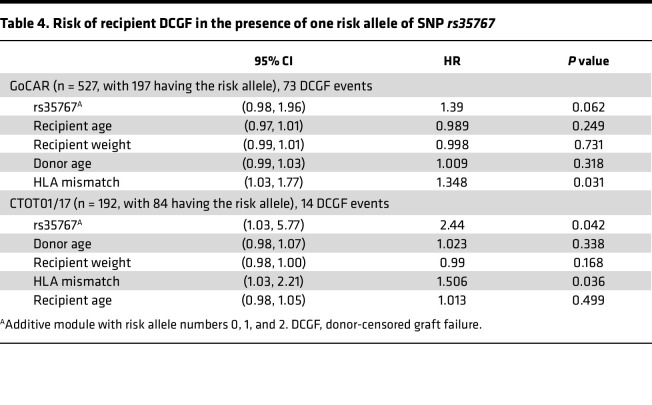
Risk of recipient DCGF in the presence of one risk allele of SNP *rs35767*

## Version 1 05/06/2025

In-Press Preview

## Version 2 06/09/2025

Electronic publication
