# Supplementary material for: The growth hormone/IGF-1 axis is a risk factor for long-term kidney allograft failure
Source: JCI Insight. 2025 May 6;10(11):e188485. doi: 10.1172/jci.insight.188485 (PMC12220966; doi:10.1172/jci.insight.188485)
Supplement: Supplemental data [file jciinsight-10-188485-s267.pdf]

Supplementary information

The Growth Hormone-IGF-1 axis is a risk factor for Long-Term Kidney Allograft Failure

Matthew Cusick[1], Viji Nair[2], Damian Fermin[2], John Hartman[2] Jeffrey A. Beamish[2], Zeguo Sun[3], Zhongyang Zhang[4], Edgar Otto[2], Rajasree Menon[2], Sudha Nadimidla[5]; Nicholas Demchuk[5]; Kelly Shaffer[5]; Peter Heeger[6], Weija Zhang[3], Madhav C. Menon[7], Matthias Kretzler[3], Roger C. Wiggins[2#] and Abhijit S. Naik[2#$]

Departments of Pathology at University of Michigan[1]; Department of Internal Medicine at University of Michigan[2], Departments of Internal Medicine Mount Sinai Hospital[3] , Genomic Sciences at Mount Sinai Hospital[4], Department of Transplant Surgery at University of Michigan[5], Cedars Sinai Hospital[6]; Department of Internal Medicine Yale University[7]

Supplemental materials provided.

Supplementary Tables

Supplementary Table 1: Relationship between IGF-1 exposure (IGF1*eKD interaction) and Death Censored Graft Failure

Supplementary Table 2: Relationship between IGF-1 exposure (IGF1*eKD interaction) and Death Censored Graft Failure also adjusted for pretransplant sensitization

Supplementary Table 3: Relationship between IGF-1 exposure (IGF1*eKD interaction) and Death Censored Graft Failure also adjusted for post-transplant donor specific antibodies (either class 1, class 2 or both)

Supplementary Table 4: Relationship between IGF-1 exposure (IGF1*eKD interaction) and Death Censored Graft Failure also adjusted for post-transplant rejection

Supplementary Table 5: Relationship between IGF-1 exposure (IGF1*eKD interaction) and proteinuria

Supplementary Table 6: Relationship between IGF-1 exposure (IGF1*eKD interaction) and Biopsy proven acute rejection.

Supplementary Table 7: Relationship between IGF-1 exposure (IGF1*eKD interaction) and Biopsy proven T cell mediated rejection.

Supplementary Table 8A: Effect of interaction between recipient race and IGF-1 on DCGF; **Table 8B**: Effect of interaction between recipient race and IGF-1 on proteinuria; **Table 8C:** Effect of interaction between recipient race and IGF-1 on TCMR; **Table 8D:** Effect of interaction between donor race and IGF-1 on DCGF; **Table 8 E**: Effect of interaction between donor race and IGF-1 on proteinuria; **Table 8F:** Effect of interaction between donor race and IGF-1 on TCMR.

Supplementary Table 9 A-C: Designated Cause of allograft loss across GoCAR, CTOT1/17 and cohort in the current study.

Supplementary Table 10: Genotype of patients with the SNP rs35767 categorized by self-reported race in the NEPTUNE Study among 620 NEPTUNE patients.

Supplementary Figures:

Supplement Figure 1: Integration of OPTN Kidney Transplant Data with Age-Specific IGF-1 Levels.

Supplementary Figure 2: Kaplan Meier failure rates for DCGF, proteinuria, Biopsy proven acute rejection (BPAR)

Supplementary Table 1: Relationship between IGF-1 exposure (IGF1*eKD interaction) and Death Censored Graft Failure

	**Haz. ratio**	**Std. err.**	**p value**	**LCL**	**UCL**
IGF1 (ng/ml)	0.99	0.00	0.10	0.99	1.00
eKD	1.01	0.01	0.33	0.99	1.04
IGF1*eKD	0.99	0.00	**0.007**	0.99	0.99
Recipient Age	0.96	0.01	0.01	0.94	0.99
Recipient Male	1.11	0.39	0.78	0.55	2.22
Recipient Race (vs. white)					
African American	3.16	1.15	0.00	1.54	6.46
Others	1.90	1.43	0.39	0.44	8.30
Donor Age	1.04	0.01	0.00	1.02	1.07
Donor Race (vs. white)					
African American	2.84	1.45	0.04	1.04	7.75
Others	3.28	1.75	0.03	1.15	9.31
Donor Male	1.18	0.41	0.62	0.61	2.32
Deceased Donor	3.51	2.18	0.04	1.04	11.88
Cold Ischemia time (min)	0.99	0.00	0.99	0.99	1.00

Abbreviations: IGF1: Insulin like growth factor 1; **eKD:** Estimated kidney dose; **LCL:** 95% lower confidence limit; **UCL:** 95% of upper confidence limit.

Supplementary Table 2: Relationship between IGF-1 exposure (IGF1*eKD interaction) and Death Censored Graft Failure also adjusted for pretransplant sensitization

	**Haz. ratio**	**Std. err.**	**p value**	**LCL**	**UCL**
IGF1	0.99	0.00	0.08	0.99	1.00
eKD	1.02	0.01	0.24	0.99	1.04
IGF1*eKD	0.99	0.00	**0.01**	0.99	0.99
Recipient Age	0.96	0.01	0.01	0.94	0.99
Recipient Male	0.91	0.35	0.81	0.43	1.92
Recipient Race (vs. white)					
African American	3.15	1.19	0.00	1.50	6.61
Others	1.49	1.13	0.60	0.33	6.61
Donor Age	1.05	0.01	0.00	1.02	1.07
Donor Race (vs. white)					
African American	3.53	1.88	0.02	1.24	10.04
Others	4.30	2.41	0.01	1.44	12.87
Donor Male	1.10	0.38	0.78	0.56	2.17
Deceased Donor	3.68	2.41	0.05	1.01	13.31
Cold Ischemia time (min)	1.00	0.00	0.80	1.00	1.00
Cpra1	0.98	0.01	0.08	0.97	1.00
Cpra2	1.00	0.01	0.73	0.98	1.02
Previous TP	0.66	0.39	0.48	0.21	2.09

Abbreviations: IGF1: Insulin like growth factor 1; **eKD:** Estimated kidney dose; TP: Transplantation; **Cpra:** Calculated panel reactive antibody; **LCL:** 95% lower confidence limit; **UCL:** 95% of upper confidence limit.

Supplementary Table 3: Relationship between IGF-1 exposure (IGF1*eKD interaction) and Death Censored Graft Failure also adjusted for post-transplant donor specific antibodies (either class 1, class 2 or both)

	Haz. ratio	Std. err.	p value	LCL	UCL
IGF1	0.99	0.00	0.08	0.99	1.00
eKD	1.01	0.01	0.48	0.98	1.04
IGF1*eKD	0.99	0.00	**0.01**	0.99	0.99
Recipient Age	0.96	0.01	0.00	0.93	0.99
Recipient Male	1.15	0.42	0.69	0.57	2.34
Recipient Race (vs. white)					
African American	2.96	1.12	0.00	1.41	6.20
Others	2.19	1.70	0.31	0.48	10.00
Donor Age	1.04	0.01	0.00	1.02	1.07
Donor Race (vs. white)					
African American	2.77	1.40	0.05	1.02	7.48
Others	3.04	1.64	0.04	1.06	8.73
Donor Male	1.15	0.40	0.69	0.58	2.27
Deceased Donor	3.62	2.30	0.04	1.05	12.55
Cold Ischemia time (min)	1.00	0.00	0.98	1.00	1.00
donor specific antibody	1.44	0.51	0.30	0.72	2.88

Abbreviations: IGF1: Insulin like growth factor 1: **eKD:** Estimated kidney dose; **LCL:** 95% lower confidence limit; **UCL:** 95% of upper confidence limit.

Supplementary Table 4: Relationship between IGF-1 exposure (IGF1*eKD interaction) and Death Censored Graft Failure also adjusted for post-transplant rejection

	Haz. ratio	Std. err.	p value	LCL	UCL
IGF1	0.99	0.00	0.18	0.99	1.00
eKD	1.01	0.01	0.54	0.98	1.04
IGF1*eKD	0.99	0.00	**0.01**	0.99	0.99
Recipient Age	0.96	0.01	0.01	0.94	0.99
Recipient Male	1.04	0.38	0.92	0.51	2.13
Recipient Race (vs. white)					
African American	4.01	1.74	0.00	1.71	9.39
Others	2.94	2.34	0.18	0.62	13.96
Donor Age	1.06	0.02	0.00	1.03	1.09
Donor Race (vs. white)					
African American	2.08	1.12	0.18	0.72	6.00
Others	1.32	0.83	0.66	0.39	4.50
Donor Male	1.02	0.38	0.95	0.49	2.14
Deceased Donor	3.28	2.22	0.08	0.87	12.37
Cold Ischemia time (min)	0.99	0.00	0.94	0.99	1.00
Type of rejection					
TCMR	5.02	2.46	0.00	1.92	13.11
AMR	6.60	4.22	0.00	1.89	23.08
Mixed	4.20	2.86	0.04	1.11	15.93

Abbreviations: IGF1: Insulin like growth factor 1: **eKD:** Estimated kidney dose; **TCMR:** T-cell mediated rejection; **AMR:** Antibody mediated rejection**; LCL:** 95% lower confidence limit; **UCL:** 95% of upper confidence limit.

Supplementary Table 5: Relationship between IGF-1 exposure (IGF1*eKD interaction) and proteinuria

	Haz. ratio	Std. err.	p value	LCL	UCL
IGF1	0.99	0.00	0.61	0.99	1.00
eKD	1.02	0.02	0.19	0.99	1.05
IGF1*eKD	0.99	0.00	**0.01**	0.99	0.99
Recipient Age	0.97	0.01	0.06	0.94	1.00
Recipient Male	0.93	0.38	0.87	0.42	2.09
Recipient Race (vs. white)					
African American	2.15	0.91	0.07	0.94	4.91
Others	1.08	0.99	0.93	0.18	6.51
Donor Age	1.03	0.01	0.07	1.00	1.05
Donor Race (vs. white)					
African American	3.93	2.24	0.02	1.28	12.01
Others	2.83	1.71	0.09	0.86	9.24
Donor Male	1.38	0.53	0.39	0.66	2.91
Deceased Donor	1.39	0.97	0.64	0.35	5.47
Cold Ischemia time (min)	1.00	0.00	0.45	1.00	1.00

Abbreviations: IGF1: Insulin like growth factor 1: **eKD:** Estimated kidney dose; **LCL:** 95% lower confidence limit; **UCL:** 95% of upper confidence limit.

Supplementary Table 6: Relationship between IGF-1 exposure (IGF1*eKD interaction) and Biopsy proven acute rejection.

	Haz. ratio	Std. err.	p value	LCL	UCL
IGF1	0.99	0.00	0.13	0.99	1.00
eKD	1.01	0.01	0.60	0.98	1.03
IGF1*eKD	0.99	0.00	0.07	0.99	1.00
Recipient Age	0.97	0.01	0.02	0.94	0.99
Recipient Male	0.82	0.32	0.61	0.39	1.74
Recipient Race (vs. white)					
African American	0.79	0.31	0.56	0.36	1.73
Others	0.74	0.50	0.66	0.20	2.75
Donor Age	0.97	0.01	0.05	0.95	1.00
Donor Race (vs. white)					
African American	6.10	3.10	0.00	2.25	16.54
Others	7.39	3.33	0.00	3.06	17.86
Donor Male	1.22	0.43	0.58	0.61	2.44
Deceased Donor	1.96	1.17	0.26	0.61	6.29
Cold Ischemia time (min)	1.00	0.00	0.82	1.00	1.00
Cpra1	1.00	0.01	0.92	0.98	1.02
Cpra2	1.01	0.01	0.61	0.99	1.02
Previous TP	0.92	0.44	0.86	0.36	2.36

Abbreviations: IGF1: Insulin like growth factor 1; **eKD:** Estimated kidney dose; **TP:** Transplantation; **Cpra:** Calculated panel reactive antibody; **LCL:** 95% lower confidence limit; **UCL**: 95% of upper confidence limit.

Supplementary Table 7: Relationship between IGF-1 exposure (IGF1*eKD interaction) and Biopsy proven T cell mediated rejection.

	**Haz. ratio**	**Std. err.**	**p value**	**LCL**	**UCL**
IGF1	0.99	0.00	0.06	0.99	1.00
eKD	1.01	0.02	0.79	0.97	1.04
IGF1*eKD	0.99	0.00	**0.04**	0.99	0.99
Recipient Age	0.96	0.02	0.02	0.92	0.99
Recipient Male	0.37	0.18	0.04	0.14	0.96
Recipient Race (vs. white)					
African American	0.46	0.27	0.19	0.14	1.46
Others	1.92	1.48	0.40	0.43	8.69
Donor Age	0.98	0.02	0.22	0.95	1.01
Donor Race (vs. white)					
African American	11.62	8.40	0.00	2.82	47.93
Others	10.05	5.12	0.00	3.71	27.26
Donor Male	1.39	0.62	0.46	0.58	3.34
Deceased Donor	3.08	2.38	0.15	0.68	14.01
Cold Ischemia time (min)	1.00	0.00	0.62	1.00	1.00
Cpra1	0.98	0.01	0.18	0.96	1.01
Cpra2	1.02	0.01	0.23	0.99	1.04
Previous TP	1.37	0.67	0.52	0.53	3.58

Abbreviations: **IGF1:** Insulin like growth factor 1: **eKD:** Estimated kidney dose; **TP:** Transplantation; **Cpra:** Calculated panel reactive antibody; **LCL:** 95% lower confidence limit; **UCL:** 95% of upper confidence limit.

Supplementary Table 8A: Effect of interaction between recipient race and IGF-1 on DCGF

	Haz. ratio	Std. err.	p value	LCL	UCL
**IGF-1**	0.99	0.002	0.31	0.99	1.002
**Recipient Race (vs. white)**					
African American	3.00	2.13	0.12	0.75	12.08
Others	53.32	99.64	0.03	1.37	2077
**Recipient race * IGF-1**					
African American	1.00	0.003	0.96	0.99	1.01
Others	0.98	0.013	0.11	0.95	1.01

Abbreviations: IGF1: Insulin like growth factor 1; **LCL**: 95% lower confidence limit; **UCL:** 95% of upper confidence limit. Models adjusted for recipient age, recipient gender, eKD, donor age, donor race, donor gender, cold ischemia time, type of kidney (deceased or living)

Supplementary Table 8B: Effect of interaction between recipient race and IGF-1 on proteinuria

	Haz. ratio	Std. err.	p value	LCL	UCL
**IGF-1**	1.00	0.002	0.46	0.998	1.01
**Recipient Race (vs. white)**					
African American	7.44	6.49	0.02	1.35	41.10
Others	192.25	470.13	0.03	1.59	23194.42
**Recipient race * IGF-1**					
African American	0.99	0.004	0.13	0.99	1.00
Others	0.97	0.02	0.09	0.93	1.01

Abbreviations: IGF1: Insulin like growth factor 1; **LCL**: 95% lower confidence limit; **UCL:** 95% of upper confidence limit. Models adjusted for recipient age, recipient gender, eKD, donor age, donor race, donor gender, cold ischemia time, type of kidney (deceased or living)

Supplementary Table 8C: Effect of interaction between recipient race and IGF-1 on TCMR

	Haz. ratio	Std. err.	p value	LCL	UCL
**IGF-1**	1.00	0.003	0.07	0.99	1.00
**Recipient Race (vs. white)**					
African American	0.66	0.86	0.75	0.05	8.50
Others	1.46	2.76	0.84	0.04	59.09
**Recipient race * IGF-1**					
African American	0.99	0.007	0.92	0.99	1.01
Others	1.00	0.008	0.95	0.99	1.02

Abbreviations: IGF1: Insulin like growth factor 1; **LCL:** 95% lower confidence limit; **UCL**: 95% of upper confidence limit. Models adjusted for recipient age, recipient gender, eKD, donor age, donor race, donor gender, cold ischemia time, type of kidney (deceased or living)

Supplementary Table 8D: Interaction between donor race and IGF-1 on DCGF

	Haz. ratio	Std. err.	p value	LCL	UCL
**IGF-1**	1.00	0.00	0.13	0.99	1.00
**Donor race (vs. white)**					
African American	4.14	6.50	0.37	0.19	89.82
Other	2.81	4.19	0.49	0.15	52.12
**Donor race* IGF1**					
African American	1.00	0.01	0.68	0.98	1.02
Other	1.00	0.01	0.93	0.99	1.01

Abbreviations: IGF1: Insulin like growth factor 1; **LCL:** 95% lower confidence limit; **UCL**: 95% of upper confidence limit. Models adjusted for recipient age, recipient gender, eKD, donor age, donor race, donor gender, cold ischemia time, type of kidney (deceased or living)

Supplementary Table 8E: Interaction between donor race and IGF-1 on proteinuria

	Haz. ratio	Std. err.	p value	LCL	UCL
**IGF-1**	1.00	0.002	0.83	0.99	1.004
**Donor race (vs. white)**					
African American	13.41	20.05	0.08	0.72	251.26
Other	33.26	55.43	0.04	1.27	871.97
**Donor race* IGF1**					
African American	0.99	0.009	0.26	0.97	1.007
Other	0.99	0.01	0.18	0.97	1.006

Abbreviations: IGF1: Insulin like growth factor 1; **LCL:** 95% lower confidence limit; **UCL**: 95% of upper confidence limit. Models adjusted for recipient age, recipient gender, eKD, donor age, donor race, donor gender, cold ischemia time, type of kidney (deceased or living).

Supplementary Table 8F: Interaction between donor race and IGF-1 on TCMR

	Haz. ratio	Std. err.	p value	LCL	UCL
**IGF-1**	0.99	0.003	0.04	0.99	1.00
**Donor race (vs. white)**					
African American	4.21	5.53	0.28	0.32	55.27
Other	4.80	5.04	0.14	0.61	37.60
**Donor race* IGF1**					
African American	1.004	0.006	0.54	0.99	1.02
Other	1.005	0.006	0.41	0.99	1.02

Abbreviations: IGF1: Insulin like growth factor-1; **LCL:** 95% lower confidence limit; **UCL**: 95% of upper confidence limit. Models adjusted for recipient age, recipient gender, eKD, donor age, donor race, donor gender, cold ischemia time, type of kidney (deceased or living).

Supplementary Table 9 A-C: Cause of allograft loss across cohorts.

A. Designated causes of graft loss in current clinical study

Death Censored Graft Failure (n=44)	CAN/CR	AR	FSGS	Infection	PNF	AKI	Others
	11 (26.2%)	2 (4.8%)	5 (11.9%)	6 (14.3%)	5 (11.9%)	6 (14.3%)	7* (16.7)

B. Designated causes of graft loss in GoCAR study

Death Censored Graft Failure (n=50)	Chronic allograft nephropathy/chronic rejection	Acute rejection	Infections	Others	Primary non function
	18 (36%)	8 (16%)	1 (7%)	11(22%)	2(4%)

Reference: Sun, Z., Zhang, Z., Banu, K., Gibson, I. W., Colvin, R. B., Yi, Z., ... & Menon, M. C. (2023). Multiscale genetic architecture of donor-recipient differences reveals intronic LIMS1 mismatches associated with kidney transplant survival. The Journal of clinical investigation, 133(21).

C. Designated causes of graft loss in the CTOT1/17 study

Death Censored Graft Failure (n=14)	Chronic allograft nephropathy/chronic rejection	Acute rejection	Infections	Others
	7 (50%)	3 (21%)	1 (7%)	3(21%)

Faddoul, G., Nadkarni, G. N., Bridges, N. D., Goebel, J., Hricik, D. E., Formica, R., ... & Heeger, P. S. (2018). Analysis of biomarkers within the initial 2 years posttransplant and 5-year kidney transplant outcomes: results from clinical trials in organ transplantation-17. Transplantation, 102(4), 673-680.

CAN/CR: Chronic allograft nephropathy/chronic rejection; FSGS (focal segmental glomerulosclerosis); PNF: Primary non function; AKI: Acute Kidney Injury

* 6/7 patients had no biopsies before graft failure (all others were biopsy proven) due to slow progressive decline in kidney function and presumed CAN/CR per the treating physician notes as has been done in previous studies. Thus, the proportion of patients with CAN/CR could be higher in alignment with the CTOT and GoCAR study.

Supplementary Table 10: Genotype of patients with the SNP rs35767 categorized by self-reported race in the NEPTUNE Study among 620 NEPTUNE patients.

Self-Reported Race	REF	HET	HOM_ALT	Total	NEPTUNE AF
Asian/Asian American	9	23	30	62	0.6694
Black/African American	19	85	43	147	0.5816
Multi-Racial	2	15	15	32	0.7031
Native American/Alaskan Native/First Nation	0	1	0	1	0.5
Native Hawaiian/Other Pacific Islander	0	2	2	4	0.75
Unknown	4	21	21	46	0.6848
White/Caucasian	9	86	233	328	0.8415

Abbreviations: REF: Reference; **HET**: Heterogenous; **HOM**: Homozygous; **ALT:** Alternate; **AF**: Allele Frequency

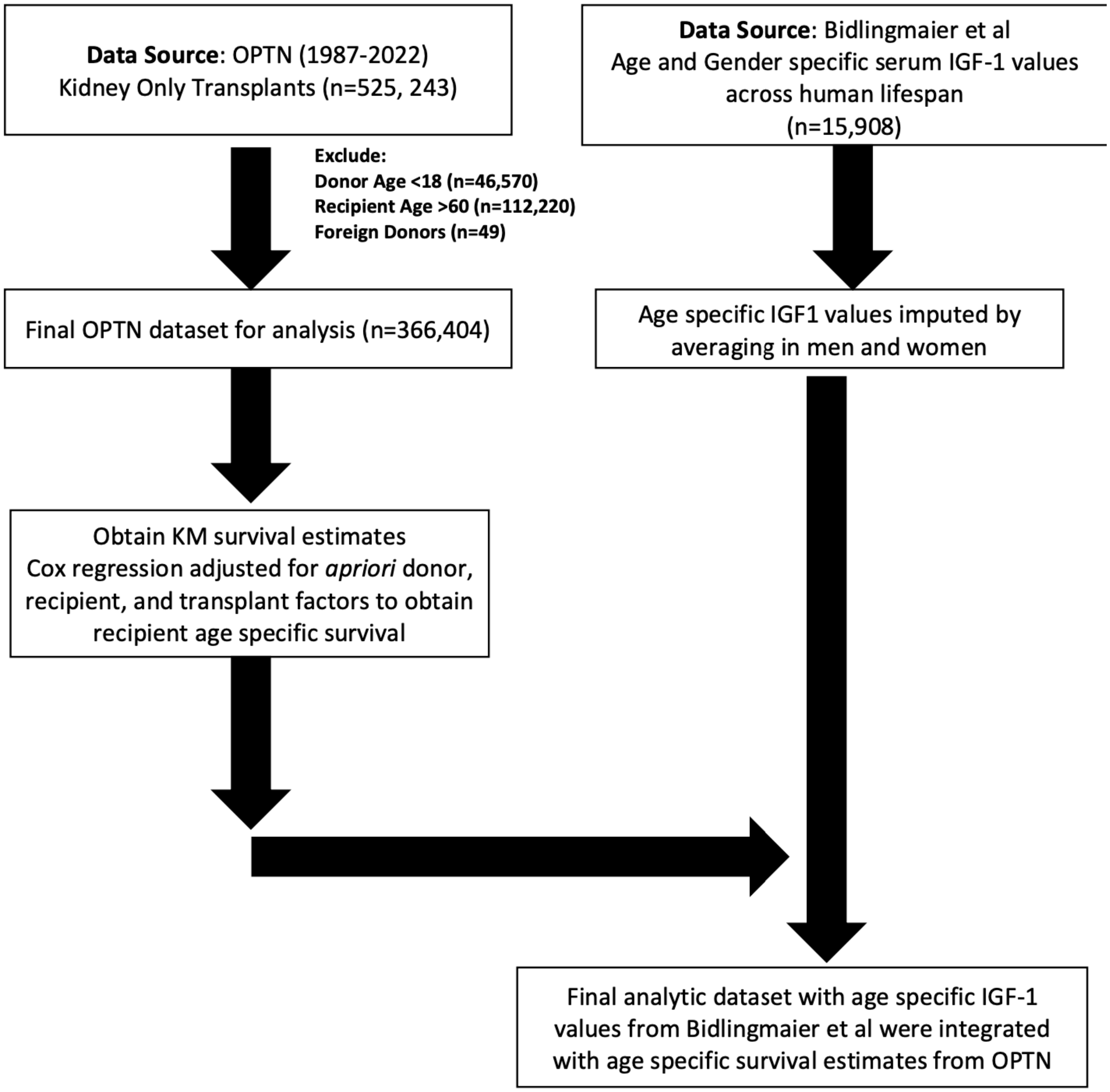

Supplement Figure 1: Integration of OPTN Kidney Transplant Data with Age-Specific IGF-1 Levels. This figure illustrates the methodology and data integration process used in our study. We analyzed the Organ Procurement and Transplantation Network (OPTN) dataset spanning from 1987 to 2022, focusing exclusively on patients who received kidney-only transplants. Initial data included 525,243 patients; after excluding donors younger than 18, recipients older than 60, and foreign donors, the cohort was narrowed down to 366,404 patients. Concurrently, age and gender-specific serum IGF-1 levels across the human lifespan were obtained from the study by Bidlingmaier et al. Average age- specific IGF-1 values were calculated by averaging data for both genders. These values were then integrated with age-specific survival estimates derived from the OPTN dataset to assess the impact of IGF-1 levels on post-transplant outcomes. This figure provides a schematic representation of the data extraction and integration processes employed to facilitate this comprehensive analysis

A. Death Censored Graft Failure

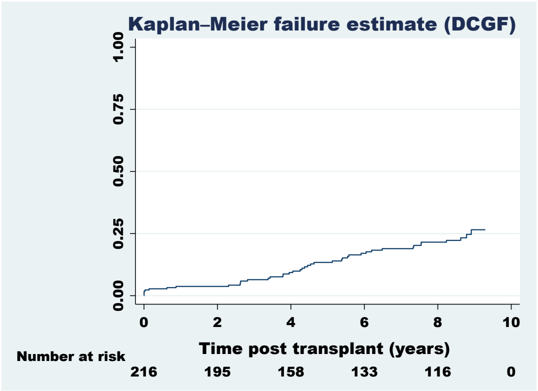

B. Proteinuria (>1 g/g)

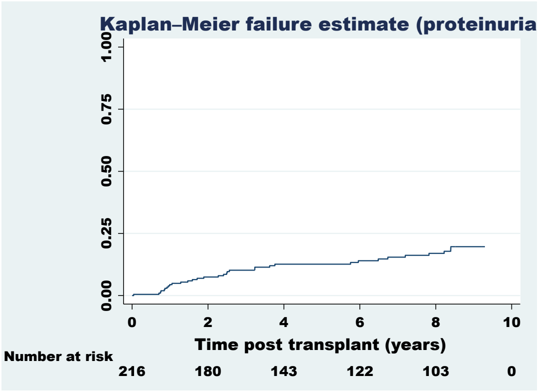

C. Biopsy Proven Acute Rejection

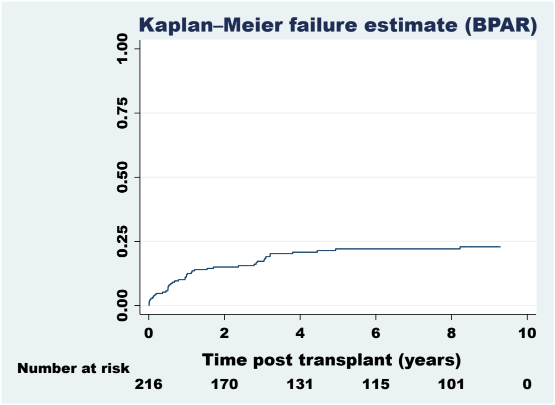

Supplementary Figure 2: Clinical cohort: Kaplan-Meier failure estimate for **A. Death Censored Graft Failure (DCGF); B. Proteinuria** and **C. Biopsy-proven acute rejection (BPAR)** along with number at risk at various time points post kidney transplantation.

Supplementary methods for single cell and pseudobulk analysis of transcripts for the GH-IGF-1 axis.

Single-cell processing of the research core: The study uses data previously published (Menon et al., 2022) (1) . The generation of single-cell preparations was accomplished by enzymatic (Liberase TL; Sigma Aldrich) and mechanical dissociation for 12 minutes at 37 °C following our previously published protocol (2) . Cells are filtered through a 30 μm strainer and counted, and up to 10,000 viable cells are submitted to the University of Michigan Advanced Genomics Core facility to execute droplet-based high-throughput scRNA-seq on the 10x Genomics Chromium platform. After droplet encapsulation, the 10x Genomics approach allows cell lysis, individual cell RNA molecular barcoding, and reverse transcription. Subsequently, cDNA libraries are generated and sequenced on the NovaSeq 6000 platform (Illumina) as asymmetric paired end (26 × 151) runs and generating >200 million raw sequence reads per sample. The sequencing data are preprocessed using the 10x Genomics software Cell Ranger. Downstream analysis was performed with the Seurat R package version 3 (Satija Lab) (3). A combined analysis of the single-cell data sets generated from the different sample sources (living donor and surveillance biopsies) using Seurat version 3 includes the following steps: filtering of cells with <500 genes or >50% mitochondrial content default normalization, scaling based on sample mRNA count and mitochondrial RNA content, dimensionality reduction principal component analysis and uniform manifold approximation and projection, sample integration using the Harmony algorithm, standard unsupervised clustering, and the discovery of differentially expressed cell type-specific markers. Cell-type specific differential gene expression analyses (DEGs) were performed using a pseudobulk approach, which was employed to mitigate the risk of false positive discovery commonly associated with single-cell transcriptomic studies (4). The signaling elements tested, determined *a priori* from the literature, included transcripts for *IGF1, IGF2*, IGF-1 receptor (*IGF1R*), growth hormone receptor (*GHR*), and IGF binding proteins (*IGFBPs 1-6*) (5).

Pseudobulking strategies: Pseudobulking strategies were applied to minimize the risk of false positive results in single-cell data, especially among cells with low counts (4) .After clustering and cell type identification, the pseudo-bulk matrices were generated from the single-cell matrix (25,720 genes as rows, 40,254 cells as columns) by first aggregating cell-wise raw counts into their corresponding cell type/sample bins using summation. These data have been previously published in Menon et al. and are available on the Gene Expression Omnibus, Accession number GSE169285 (1) . From 34 samples and 29 cell types, this yielded a matrix with 965 cell type/sample columns, excluding 31 representing cases where cell types did not occur in a given sample. To facilitate cell-type-specific analysis over samples, 29 cell-type-specific matrices, each with genes as rows and samples as columns, were extracted along corresponding sample-related metadata. This process conserved the number of counts in the original single-cell data exactly, leaving all QC-related filtering of genes and samples to downstream analysis. Generally, as some cell types are much more abundant than others – due both to their varied frequency in the original biopsy samples and recovery biases in the single-cell protocol itself (e.g., from dissociation efficiencies) – and sequencing coverage per cell also varies widely, some samples in some cell types were of insufficient quality (sequencing depth) to support analysis and were excluded. For differential expression analysis, a sample was excluded in a particular cell type if it was supported by fewer than 10 cells or 10,000 total counts. At this point, each cell type-specific counts matrix could be treated similarly to one derived from bulk RNA-seq, with normalization done using limma voom and differential expression with limma.

Reference for Supplementary Methods.

1. Hosmer DW, Lemeshow S, and May S. Applied survival analysis. *Wiley Series in Probability and Statistics.* 2008:60.
2. Arazi A, Rao DA, Berthier CC, Davidson A, Liu Y, Hoover PJ, et al. The immune cell landscape in kidneys of patients with lupus nephritis. *Nature immunology.* 2019:1.
3. Butler A, Hoffman P, Smibert P, Papalexi E, and Satija R. Integrating single-cell transcriptomic data across different conditions, technologies, and species. *Nat Biotechnol.* 2018;36(5):411-20.
4. Squair JW, Gautier M, Kathe C, Anderson MA, James ND, Hutson TH, et al. Confronting false discoveries in single-cell differential expression. *Nature Communications.* 2021;12(1):5692.
5. Bach LA. 40 years of IGF1: IGF-binding proteins. *Journal of molecular endocrinology.* 2018;61(1):T11-T28.
